# Comparison of viral RNA–host protein interactomes across pathogenic RNA viruses informs rapid antiviral drug discovery for SARS-CoV-2

**DOI:** 10.1038/s41422-021-00581-y

**Published:** 2021-11-04

**Authors:** Shaojun Zhang, Wenze Huang, Lili Ren, Xiaohui Ju, Mingli Gong, Jian Rao, Lei Sun, Pan Li, Qiang Ding, Jianwei Wang, Qiangfeng Cliff Zhang

**Affiliations:** 1 MOE Key Laboratory of Bioinformatics, Beijing Advanced Innovation Center for Structural Biology & Frontier Research Center for Biological Structure, Center for Synthetic and Systems Biology, Tsinghua-Peking Joint Center for Life Sciences, School of Life Sciences, Tsinghua University, Beijing, China; 2 NHC Key Laboratory of Systems Biology of Pathogens and Christophe Mérieux Laboratory, Institute of Pathogen Biology, Chinese Academy of Medical Sciences & Peking Union Medical College, Beijing, China; 3 Key Laboratory of Respiratory Disease Pathogenomics, Chinese Academy of Medical Sciences and Peking Union Medical College, Beijing, China; 4 Center for Infectious Disease Research, School of Medicine, Tsinghua University, Beijing, China

**Keywords:** Proteomics, Cell biology

## Abstract

In contrast to the extensive research about viral protein–host protein interactions that has revealed major insights about how RNA viruses engage with host cells during infection, few studies have examined interactions between host factors and viral RNAs (vRNAs). Here, we profiled vRNA–host protein interactomes for three RNA virus pathogens (SARS-CoV-2, Zika, and Ebola viruses) using ChIRP-MS. Comparative interactome analyses discovered both common and virus-specific host responses and vRNA-associated proteins that variously promote or restrict viral infection. In particular, SARS-CoV-2 binds and hijacks the host factor IGF2BP1 to stabilize vRNA and augment viral translation. Our interactome-informed drug repurposing efforts identified several FDA-approved drugs (e.g., Cepharanthine) as broad-spectrum antivirals in cells and hACE2 transgenic mice. A co-treatment comprising Cepharanthine and Trifluoperazine was highly potent against the newly emerged SARS-CoV-2 B.1.351 variant. Thus, our study illustrates the scientific and medical discovery utility of adopting a comparative vRNA–host protein interactome perspective.

## Introduction

The SARS-CoV-2 coronavirus is the causal pathogen of the ongoing Coronavirus Disease 2019 (COVID-19) pandemic, resulting in more than 228 million infections and 4 million deaths and global disruption of society and economy.^1,2^ SARS-CoV-2 is an RNA virus which relies heavily on interactions with host factor biomolecules to complete its life cycle.^3^ RNA-binding proteins function in many aspects of cellular and viral processing, e.g., RNA translation, stabilization, modification, and localization.^4,5^ Many studies have focused on characterization of viral protein–host protein interactions.^6–10^ In contrast, interactions between host proteins and viral RNA (vRNA) are much less well understood, despite the known importance of the viral RNA genome for multiple processes during infection, including viral genome translation and replication.^11^ Recent years have seen an explosion in high-throughput methods that enable global analyses of RNA–protein interactions (“the interactome”) in cells.^12,13^ These approaches can substantially advance our understanding of the infection and pathology of RNA viruses and can inform diverse and effective therapeutic options.

Here, we unveiled the vRNA–host protein interactomes for SARS-CoV-2 and two other dangerous RNA viruses: Ebola virus (EBOV)^14^ and Zika virus (ZIKV).^15^ Our ChIRP-MS (comprehensive identification of RNA-binding proteins by mass spectrometry)^16^ analyses in infected human host cells revealed many functional host factors and interaction patterns that reflect both common and virus-specific host responses. We applied the insights from our comparative interactome datasets to inform a targeted antiviral drug screening workflow based on repurposing of FDA-approved drugs. Ultimately, we showed that selective inhibition of host vRNA-binding proteins can attenuate infection and potently inform development of innovative therapies for viral infections including COVID-19.

## Results

### ChIRP-MS reveals vRNA–host protein interactomes for SARS-CoV-2, Zika, and Ebola viruses

**Fig. 1:**
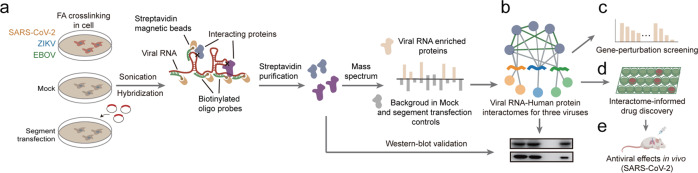
Schematic for discovery of vRNA-interacting proteins and functional host factors for SARS-CoV-2, ZIKV and EBOV. **a** ChIRP-MS was performed to identify human proteins that interact with viral RNA genomes in cells infected with the indicated RNA viruses. Human cells were infected with SARS-CoV-2, ZIKV or EBOV. Two types of control were used to specifically enrich vRNA-interacting proteins in infected samples: mock (cells without infection) and the “segment transfection” (cells expressing vRNA segments of the SARS-CoV-2, ZIKV, or EBOV genomes by plasmid transfection). Virus-infected, mock, and vRNA segment-transfected cells were crosslinked using formaldehyde and then sonicated to release RNA–protein complexes. For each virus, the vRNA–human protein complexes were purified using biotinylated oligos specifically tiling the viral RNA genome, and the co-purified human proteins were identified using mass spectrometry. FA, formaldehyde. **b** Comparison of the different vRNA interactomes identified the common and the virus-specific interactors. **c** Gene loss-of-function screen for functional interactors that affect virus infection. **d** Antiviral drug discovery informed by the comparative interactomes. We developed an antiviral drug screening workflow based on repurposing of FDA-approved drugs that targeting the vRNA-interacting proteins. Then the antiviral activities of the repurposing drugs against the infections of SARS-CoV-2, ZIKV, and EBOV were experimentally confirmed in this study. **e** Focusing on COVID-19, antiviral activities of the selected repurposing drugs were evaluated in a mouse model challenged with SARS-CoV-2.

To discover the vRNA–host protein interactomes for SARS-CoV-2, EBOV, and ZIKV, we performed ChIRP-MS in virus-infected human host cells, using mock (without virus infection) and vRNA segment transfection samples as controls (Fig. 1a; Materials and Methods). Comparison of the different vRNA interactomes reflects both common and virus-specific host responses (Fig. 1b). We screened for functional interactors using gene loss-of-function experiments (Fig. 1c). We then applied the insights from our comparative interactome datasets to inform a targeted antiviral drug screening workflow based on repurposing of FDA-approved drugs, and experimentally identified antiviral compounds targeting the vRNA interactors that inhibit the infections of SARS-CoV-2, ZIKV, and EBOV (Fig. 1d). Finally, focusing on COVID-19, we identified drugs as broad spectrum antivirals that repress the infection of SARS-CoV-2 in vivo (Fig. 1e).

**Fig. 2:**
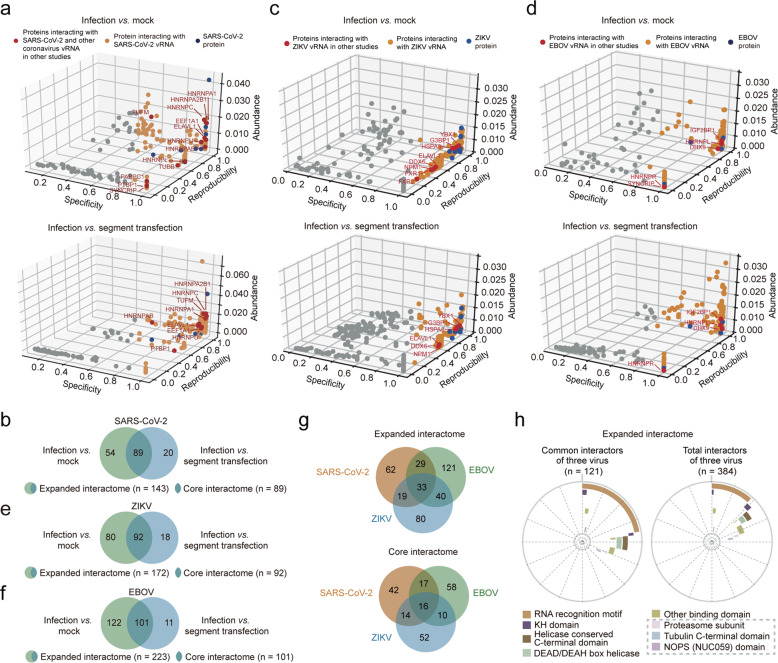
Identification of the human proteins interacting with the SARS-CoV-2, ZIKV, and EBOV RNA genome in infected cells. **a**, **c**, **d** Evaluation of the SARS-CoV-2 (**a**), ZIKV (**c**), and EBOV (**d**) vRNA-interacting proteins identified by ChIRP-MS using the “mock” control (top) and the “segment transfection” control (bottom). Proteins previously known to interact with other coronavirus RNA/ZIKV/EBOV genomes are indicated as red dots; otherwise as orange dots; SARS-CoV-2/ZIKV/EBOV viral proteins co-purified with vRNA are indicated as blue dots. Protein abundance in virus-infected samples (Abundance), protein abundance over different replicates (Reproducibility), and uniqueness of an interacting protein across all replicated experiments (Specificity) were evaluated to define the vRNA-interacting proteins (see Materials and Methods). **b**, **e**, **f** Comparison of vRNA-interacting proteins of SARS-CoV-2 (**b**), ZIKV (**e**), and EBOV (**f**) identified using the “mock” or the “segment transfection” controls. Infection vs mock, vRNA-interacting proteins enriched by using the “mock” control, defined as the “expanded interactome”. Infection vs segment transfection, vRNA-interacting proteins enriched by using the “segment transfection” control. The overlapped proteins were defined as the “core interactome”. **g** Comparison of common interacting proteins between the expanded vRNA interactomes (up) or the core interactomes (bottom) for SARS-CoV-2, ZIKV, and EBOV. See also **b**, **e**, **f**. **h** Distribution of enriched protein domains among the common (left) and total (right) interactors of three viruses in the expanded interactomes for SARS-CoV-2, ZIKV, and EBOV. Bold lines with different colors represent different protein domains. Protein domains not known as RNA-binding domains are indicated using gray dashed boxes.

To define host proteins associated with genomic RNA of SARS-CoV-2 (IPBCAMS-YL01/2020), we used the RNA-directed proteomic discovery method ChIRP-MS^16^ (Materials and Methods). We used human hepatocarcinoma Huh7.5.1 cells, which are permissive for virus replication,^17^ for our study. We also optimized the multiplicity of infection (MOI) and time-point of sample harvest to permit amplification of viral genomic RNA to levels that would support binding of sufficient protein levels for mass spectrometry-based identification. Specifically, Huh7.5.1 cells were infected with SARS-CoV-2 (MOI 0.05) for 30 h, a point when only limited virus-induced cell death was observed and when the vRNA levels were close to the endogenous *GAPDH* RNA level (Supplementary information, Fig. S1a); this enabled us to identify as comprehensive a set of vRNA-interacting proteins as possible. The infected cells were then crosslinked with formaldehyde to preserve vRNA–protein complexes. Biotinylated oligonucleotides specifically tiling SARS-CoV-2 vRNA were used to enrich vRNA–host protein complexes from cell lysates (Supplementary information, Table S1) and the co-purified proteins were identified by mass spectrometry (Fig. 2a). We calculated the enrichment of co-purified proteins for the “virus infection” samples over the “mock” control samples (cells without virus infection), and defined the list of enriched proteins as the “expanded” interactome. We also calculated the enrichment of co-purified proteins for the “virus infection” samples over the “segment transfection” control samples, and then intersect the resulting enriched protein list with the “expanded” interactome to define the “core” interactome (Materials and Methods).

Our expanded interactome of SARS-CoV-2 comprised a total of 143 human proteins (Fig. 2a, up; Supplementary information, Table S2). The co-purification steps of the ChIRP-MS strategy depleted highly abundant host RNAs while still enabling robust recovery of over 80% total vRNA and 70% genomic RNA of SARS-CoV-2 in infected cells (Supplementary information, Fig. S1b), suggesting that we identified interactors of SARS-CoV-2 positive-strand RNA without obvious bias. Correlation coefficients across three biological replicates were all above 0.9 (Supplementary information, Fig. S1c, d). The expanded interactome underscored the extensive physical associations between vRNA and the SARS-CoV-2 nucleocapsid protein (NP), the viral M protein, the spike (S) protein, and several nonstructural proteins (NS) (Fig. 2a, up). Our final core interactome contains 89 proteins (Fig. 2a, bottom, b). The segment transfection method enriches for proteins specifically recruited by vRNA during infection, so the proteins of the core interactome should be more likely to functionally impact the SARS-CoV-2 life cycle than the expanded interactome proteins.

SARS-CoV-2 attacks human lung tissue and causes a respiratory disease. Our ChIRP-MS analysis in liver-derived cells may therefore not adequately mimic protein–RNA interactions in lung cells.^18^ However, interestingly, we observed significant elevations in the expression of the SARS-CoV-2 vRNA-binding proteins, specifically in lung,^19^ intestine,^20^ and brain^21^ (Supplementary information, Fig. S1e).

Seeking to delineate which host proteins are common or specific to different RNA viruses, we further used ChIRP-MS to characterize the expanded and core interactomes for ZIKV (MR766 strain) and a recombinant EBOVΔVP30-GFP virus (for which the VP30-coding sequence of the Zaire Mayinga Ebola strain was replaced by GFP)^22^ (Materials and Methods; Supplementary information, Table S1). These ChIRP-MS analyses defined a core interactome of 92 proteins and an expanded interactome of 172 proteins in ZIKV-infected Huh7 cells, and a core interactome of 101 proteins and an expanded interactome of 223 proteins in EBOVΔVP30-GFP-infected Huh7.5.1-VP30 cells (Fig. 2c–f; Supplementary information, Fig. S1f–k and Table S2).

### Confirmation of vRNA–host protein interactions in infected cells

To validate the vRNA-interacting host proteins, we assessed the ChIRP samples using western blotting (ChIRP-WB) (Materials and Methods) with antibodies for 11 proteins of the expanded interactome that were available in our lab (e.g., HnRNPU and PPIA with a high MiST score 1, and RACK1 with a lower MiST score 0.68) (Supplementary information, Fig. S2a). This analysis confirmed that 9 of the 11 tested proteins do interact with SARS-CoV-2 vRNA in infected Huh7.5.1 cells. For the expanded interactome, we compared our 143 SARS-CoV-2 vRNA binding proteins with host factors previously reported to bind coronaviruses, and found that 13 of the host factors in our ChIRP-MS interactome are known to bind the vRNA of other coronaviruses and to impact viral replication,^23,24^ among which ELVAL1, hnRNPU, and PTBP1 were confirmed by our ChIRP-WB (Fig. 2a, up; Supplementary information, Fig. S2a). Further, comparisons with two recently reported vRNA interactomes for SARS-CoV-2 based on similar RNA–protein interaction profiling methods^25,26^ revealed 17 and 46 host factors shared with our interactome dataset (Supplementary information, Fig. S2b). However, only three host proteins are shared between our vRNA-interacting proteins and a previously reported viral protein–host protein interactome,^8^ highlighting the complementary nature of the two types of interactome data (Supplementary information, Fig. S2c).

We also used ChIRP-WB to verify six of the host proteins uncovered by ChIRP-MS for their interactions with ZIKV and EBOV (IGF2BP1, ALYREF, SFPQ, MATR3, PDIA6, and SND1) (Supplementary information, Fig. S2d). Excepting an interaction between EBOV vRNA and SND1, our validation results were consistent with the ChIRP-MS results. We then examined 12 host proteins previously shown to interact with various ZIKV strains: eight of these proteins were in our expanded interactome for ZIKV, suggesting a very high sensitivity for our ChIRP-MS experiments (Fig. 2c, up; Supplementary information, Table S2; *P* < 0.001, Fisher’s exact test). Additionally, comparison against previously reported ChIRP-MS-based studies of host proteins that interact with another ZIKV strain^12^ also revealed a high level of overlap (Supplementary information, Fig. S2e; *P* < 0.001). We also examined proteins previously reported to interact with EBOV vRNA (DHX9, hnRNPR, hnRNPL, SYNCRIP, IGF2BP1),^27^ and confirmed that all these interactions are covered in our EBOV ChIRP-MS interactome (Fig. 2d, up; Supplementary information, Table S2). These analyses and observations together support that our ChIRP-MS analyses have reliably captured host proteins which interact with SARS-CoV-2, ZIKV, and EBOV vRNAs in infected cells.

To search for more potential direct binders of vRNA, we intersected our interactome proteins with RNA-binding proteins identified by the RNA interactome capture (RIC) technology in various human cell lines.^28,29^ We found that 108 (76%), 158 (92%), and 144 (65%) of SARS-CoV-2-, ZIKV-, and EBOV-interacting proteins overlapped with RIC-identified proteins, respectively (Supplementary information, Fig. S2f–h). These results indicate that many of our interactome proteins are likely direct binders.

### An integrated analysis of the three RNA virus host factor interactomes identifies common and virus-specific host factors

Comparisons amongst our expanded interactome datasets showed that a total of 33 human proteins interacted with vRNAs from all three viruses, and 88 proteins interacted with vRNAs of two of the three viruses, suggesting the potential involvement of common host factors and host reponses to infection by RNA viruses (Fig. 2g, up). One notable trend was that, compared to the total set of all vRNA-interacting proteins, the host factors common to multiple RNA viruses exhibit stronger enrichment for canonical RNA-binding domains (e.g., the RNA recognition motif, KH domain) (Fig. 2h; Supplementary information, Table S2), and showed enrichment for RNA-related functions such as translation and decay (Supplementary information, Fig. S2i, left). We next compared the core interactomes of the three RNA viruses and found that only 57 proteins are common to at least two different vRNAs (including 16 shared by all three viruses, and other 17 + 14 + 10 = 41 shared by only two viruses), while 42 are SARS-CoV-2-specific, 52 are ZIKV-specific, and 58 are EBOV-specific (Fig. 2g, bottom). Compared to the total set of core vRNA-interacting proteins, these 57 common interacting proteins also showed enrichment of RNA-related functions (Supplementary information, Fig. S2i, right).

**Fig. 3:**
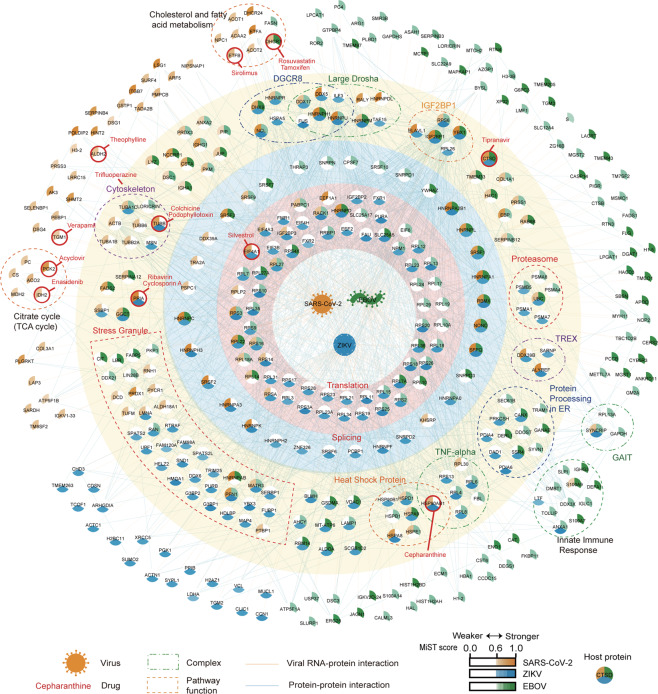
A compendium of viral RNA‒human protein interactomes for SARS-CoV-2, EBOV, and ZIKV. Central nodes represent vRNAs. Circular nodes represent host proteins. Edges indicate RNA–protein interactions (RPI, orange) and protein–protein interactions (PPI, blue). Protein complexes based on the CORUM database and proteins associated with the same biological processes according to their functional annotations are enclosed within dashed lines. Nodes are colored according to the MiST scores of proteins interacting with SARS-CoV-2 (orange), ZIKV (blue), and EBOV (green) vRNA. Drugs targeting or predicted to target the indicated proteins (Materials and Methods) are highlighted with red circles.

We further searched for common protein complexes presented in at least two of the three virus interactomes (Fig. 3; Supplementary information, Table S3; Materials and Methods). This analysis identified many common protein complexes, including the ribosome (and related translation regulators such as eIF4A), the spliceosome (and also other RNA-processing proteins such as YBX1 and RBMX), the IGF2BP1-associated complex, the small RNA-processing large Drosha and DGCR8 complexes, and the inflammatory signaling TNF-alpha/NF-κB complex, the proteasome, microtubules, and many stress granule-related proteins.

We also identified many protein complexes and pathways that were specific to only one of the three examined RNA viruses (Fig. 3; Supplementary information, Fig. S3a–c and Table S3). For example, the SARS-CoV-2 vRNA interactome was specifically enriched for host factors with annotated functions in the TCA cycle and the 2-Oxocarboxylic acid metabolism (FDR < 0.05); both of these metabolic pathways are known to be disrupted upon SARS-CoV-2 infection.^30^ ZIKV interacts with components of the TREX transcription/export complex (comprising THOCs, ALYREF, DDX39B and SARNP, Fig. 3), which functions in RNA transport, and disruption of this complex has been implicated in the ZIKV-associated disease microcephaly.^31,32^ EBOV specifically interacts with a set of immune and inflammatory responses-related proteins, including S100A9, ANXA1, and DDX3X^33–35^ and the “IFN-γ-activated inhibitor of translation” (GAIT) complex (Fig. 3), which can restrict vRNA translation.^36^

### Screening for vRNA interactors that impact virus infection

**Fig. 4:**
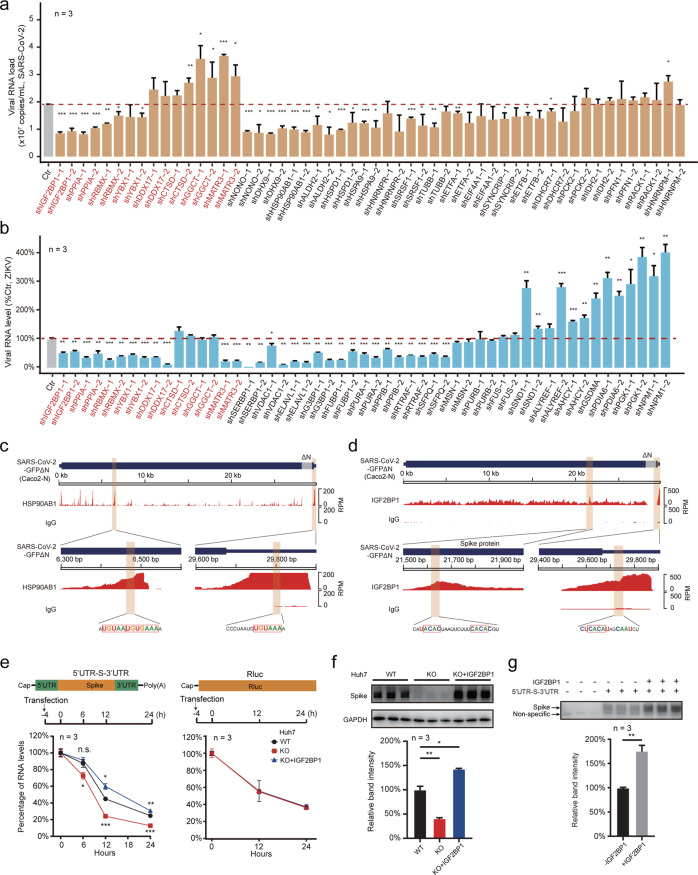
Functional characterization of vRNA-interacting proteins that impact viral infectivity. **a**, **b** Loss-of-function screen to identify functional vRNA-interacting proteins. Candidate genes of vRNA-interacting proteins were knocked down using two distinct shRNAs in Caco-2 cells (Supplementary information, Fig. S4a) and subsequently infected with SARS-CoV-2 virus (MOI 0.05) (**a**); or knocked down in Huh7 cells (Supplementary information, Fig. S4b) and subsequently infected with ZIKV MR766 virus (MOI 0.5) (**b**). For **a**, viral loads in culture supernatants were quantified at 48 h post infection by qPCR (vRNA copy numbers). For **b**, intracellular ZIKV RNA levels in the gene-knockdown cells were quantified as a percentage relative to the control samples (ZIKV-infected Ctr cells with nontargeting scramble shRNAs) at 48 h post infection by qPCR, using *GAPDH* as internal control. Host candidates knocked down in both Caco-2 and Huh7 cells are indicated in red. Data are means ± SD, *n* = 3 independent biological samples. ***P < 0.001, **P < 0.01, *P < 0.05. Two-tailed student’s *t*-test. **c**, **d** uvCLAP data for HSP90AB1 and IGF2BP1, aligned to the SARS-CoV-2 genome. HSP90AB1 (**c**) and IGF2BP1 (**d**) uvCLAP sequencing data are plotted across the SARS-CoV-2 genome. Zoomed-in views of the specific binding sites on the SARS-CoV-2 genomic RNA are shown below. The HSP90AB1-binding UA-rich sequences (**c**) and sequences matched to the known IGF2BP1-binding motif “CACA” (**d**) are highlighted and indicated in red rectangles. HSP90AB1, uvCLAP sequencing data for HSP90AB1; IGF2BP1, uvCLAP sequencing data for IGF2BP1; IgG, sequencing data of the IgG pull-down. **e** SARS-CoV-2 vRNA decay assay in Huh7 cells. Top, schematic diagram of the construction of the SARS-CoV-2 vRNA reporter (left) and the IGF2BP1 nonbinding control (right, Rluc). Middle, the schedules for RNA transfection and sample collection. The vRNA containing the 5′UTR, the coding sequence of the S protein, and the 3′UTR of SARS-CoV-2 (5′UTR-S-3′UTR) were transfected into WT cells, IGF2BP1-KO cells, and IGF2BP1 KO cells reexpressing IGF2BP1 (KO + IGF2BP1). The 5′UTR-S-3′UTR or Rluc RNA levels were quantified relative to *GAPDH* by qPCR at different hours post transfection; the percentage relative to 0 h post transfection cells is shown. **f** Detection of the S protein in WT, IGF2BP1-KO and KO + IGF2BP1 Huh7 cells. As in **e**, 5′UTR-S-3′UTR RNA of SARS-CoV-2 was transfected into cells. S protein levels in cells were detected using western blotting at 24 h post transfection. The upper panel shows a western blot for the S protein, GAPDH was used as sample loading control. Spike, western blot of S protein. The bar plot shows quantification of the band intensity from the western blot image. **g** In vitro translation assay of 5′UTR-S-3′UTR RNA. The 5′UTR-S-3′UTR RNA (500 ng) of SARS-CoV-2 was translated in rabbit reticulocyte lysates. Lysates without the 5′UTR-S-3′UTR RNA were used as a blank control for S protein detection in the in vitro translation system. Approximately 200 ng of purified IGF2BP1 was added into the lysates (+ IGF2BP1), or an equal volume of the buffer used with IGF2BP1 (–IGF2BP1). Non-specific, nonspecific protein band of the western blot. The bar plot shows quantification of the western blot. For **e**–**g**, n.s., not significant. ***P < 0.001, **P < 0.01, *P < 0.05. Two-tailed student’s *t*-test. Data are means ± SD, *n* = 3 biologically independent samples.

To help characterize the vRNA-related functions of host factors, we next generated a set of stable gene-knockdown Caco-2 and Huh7 cells using short hairpin RNA (shRNA), and then infected these with SARS-CoV-2 (MOI 0.05) or ZIKV MR766 strain (MOI 0.5). A total of 27 genes were stably knocked down in both Caco-2 and Huh7 cells (Supplementary information, Fig. S4a, b and Table S4). Infection assays revealed 2/27 anti-viral and 10/27 pro-viral host factors for SARS-CoV-2 infection in Caco-2 cells, and 7/27 anti-viral and 15/27 pro-viral host factors for ZIKV infection in Huh7 cells (Fig. 4a, b). Of particular note, knockdown of IGF2BP1-associated proteins (IGF2BP1 and YBX1)^37^ reduced the vRNA levels for both SARS-CoV-2 and ZIKV (Fig. 4a, b), as did knockdown of PPIA, a protein known to bind with vRNA during infection.^38^ Some proteins exerted opposing trends for the two viruses. For example, knockdown of MATR3 reduced ZIKV vRNA levels but increased SAR-CoV-2 vRNA levels.

We next investigated how host factors physically engage in vRNA regulation by performing ultraviolet crosslinking and affinity purification (uvCLAP, a CLIP-like technology; see Materials and Methods)^39^ using a recently reported trans-complementation system that supports SARS-CoV-2 infection and replication (Supplementary information, Fig. S4c). Briefly, this system uses a SARS-CoV-2-GFPΔΝ genome wherein the N protein-coding sequence has been replaced with GFP; the SARS-CoV-2-GFPΔΝ virions can only amplify in cells that stably express the SARS-CoV-2 N protein, thus enabling investigations in BSL-2 labs.^40^ Knockdown of heat shock proteins (HSP90AB1, HSPA9, and HSPD1) reduced the vRNA levels of SARS-CoV-2 in Huh7.5.1 cells. We confirmed that HSP90AB1 can bind SARS-CoV-2-GFPΔΝ vRNA, and observed enriched binding peaks for HSP90AB1 within the SARS-CoV-2 genome, which contains the known UA-rich HSP90AB1-binding motif^41^ (Fig. 4c).

### IGF2BP1 directly binds the SARS-CoV-2 RNA genome and promotes translation

We were particularly interested in IGF2BP1, which was among the few host factors that are common interactors to all three viruses, and was found to be able to enhance translation of hepatitis C virus RNAs (HCV) by binding to HCV UTRs.^42^ Moreover, we found that SARS-CoV-2 infection stimulates *IGF2BP1* mRNA expression both in cell lines and the lungs of COVID-19 patients (Supplementary information, Fig. S4d, e). We observed that IGF2BP1 knockdown significantly reduced the vRNA levels both of SARS-CoV-2 and ZIKV in Caco-2 and Huh7 cells, respectively (Fig. 4a, b). We also confirmed that IGF2BP1 knockout (KO) significantly repressed both SARS-CoV-2 (MOI 0.05) and ZIKV (MOI 0.5) infections in IGF2BP1-KO Huh7 cells (Supplementary information, Fig. S4f, g). In contrast, we observed no impact from IGF2BP1 knockdown on EBOV infection in experiments using a trans-complementation system with the EBOV∆VP30-GFP virus in Huh7.5.1-VP30 cells (Supplementary information, Fig. S4h).

Our uvCLAP analysis showed that IGF2BP1 bound on the genomes of SARS-CoV-2 and ZIKV, with several highly enriched binding sites including a 3′UTR site for both SARS-CoV-2 and ZIKV, and a peak within the S protein-coding region adjacent to the start codon for SARS-CoV-2 (Fig. 4d). Our data also revealed the known “CACA” motif^43^ within the binding peaks for both SARS-CoV-2 and ZIKV (Fig. 4d; Supplementary information, Fig. S4i).

Further pursuing the specific infection-related functions of IGF2BP1 binding, we constructed a viral RNA reporter system that mimics a subgenomic RNA encoding the SARS-CoV-2 S protein (5′UTR-S-3′UTR) containing the IGF2BP1-binding sites (Fig. 4e; Materials and Methods). Briefly, we transfected this 5′UTR-S-3′UTR RNA into Huh7 wild type (WT), IGF2BP1-KO cells, and IGF2BP1-KO cells in which IGF2BP1 was reexpressed (Supplementary information, Fig. S4j). Quantification using qPCR showed that the decay rate of the 5′UTR-S-3′UTR RNA was faster in IGF2BP1-KO cells (~80% vs 50% in WT) within 12 h post transfection, and the reexpression of IGF2BP1 in KO cells (KO + IGF2BP1) rescued the RNA decay rate to a similar level as that in WT cells (Fig. 4e, left). However, there were no significant differences for the decay rates of the Renilla luciferase RNA (Rluc) without IGF2BP1 binding (Fig. 4e, right). These results collectively support that IGF2BP1 functions to stabilize SARS-CoV-2 vRNA in Huh7 cells. We also observed that the level of translation products of 5′UTR-S-3′UTR (the S protein) was decreased in KO cells (Fig. 4f), and that the addition of purified IGF2BP1 to the samples increased the S protein level by in vitro translation assays using Rabbit reticulocyte lysates (Fig. 4g), both indicating that IGF2BP1 promotes translation of this SARS-CoV-2 RNA.

To test whether IGF2BP1 also regulates ZIKV vRNA, we transfected the genomic RNA of an RdRp-deficient ZIKV^44^ into IGF2BP1-KO and WT Huh7 cells. Different from the 5′UTR-S-3′UTR RNA of SARS-CoV-2, ZIKV genomic RNA did not show significant differences in RNA degradation rates in WT vs IGF2BP1-KO cells (Supplementary information, Fig. S4k), suggesting that IGF2BP1 does not function in stabilizing ZIKV genomic RNA in Huh7 cells. Experiments using a similar rabbit reticulocyte lysate assay system showed that IGF2BP1 did promote the translation of ZIKV RNA. In contrast, translation of Rluc RNA with no IGF2BP1-binding site was not enhanced by adding the IGF2BP1 protein (Supplementary information, Fig. S4l).

### Interactome-informed drug repurposing discovered potent and broad-spectrum antiviral compounds

Existing drugs that are known to target various host proteins can be exploited for potential antiviral treatments.^8^ Given the urgent need for COVID-19 therapies,^45^ we analyzed open-source chemical databases (e.g., Drugbank, Drugcentral, ChEMBL, etc.) to find compounds that target host factors we identified in our SARS-CoV-2 vRNA interactome. Among a total of 5309 compounds (Supplementary information, Table S5) that are known or predicted to interact with 56 SARS-CoV-2 vRNA-binding proteins, we prioritized approved drugs and clinical phase agents that were available at the Center of Pharmaceutical Technology of Tsinghua University, and examined the antiviral activities of candidate drugs against SARS-CoV-2, ZIKV, and EBOV (Supplementary information, Fig. S5a and Table S6).

Our initial assessment of the potential antiviral effects of 21 of these drugs against SARS-CoV-2 infection used the aforementioned SARS-CoV-2-GFPΔN trans-complementation system (Supplementary information, Fig. S4c), wherein GFP expression is used to monitor viral infection and replication in human cells. The initial screen using 10 µM of each selected compound uncovered activity against SARS-CoV-2-GFPΔN for 15 of the 21 tested candidate drugs. Compared with the DMSO vehicle control, five exert strong antiviral effects (Supplementary information, Fig. S5b).

**Fig. 5:**
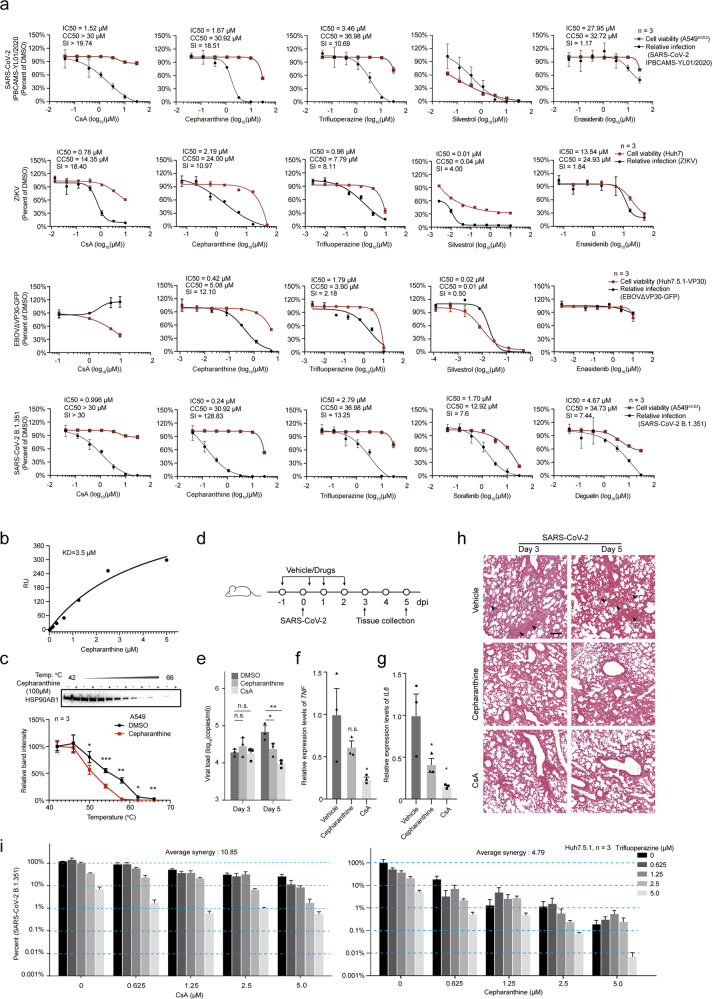
Validation of antiviral activities of FDA-approved and clinical-trial drugs targeting vRNA-interacting proteins. **a** The antiviral activities of the indicated drugs against infection with SARS-CoV-2 (IPBCAMS-YL01/2020) (first row), ZIKV (MR766) (second row), EBOVΔVP30-GFP (third row), and the B.1.351 SARS-CoV-2 variant (fourth row), at different drug concentrations. Red line, cell viability; black line, infection ratio relative to the vehicle control (DMSO) group. Cell lines used for infection assay were indicated on the right. Data are means ± SD. *n* = 3 biologically independent samples. IC50, CC50 and SI values are indicated. **b** Surface Plasmon Resonance (SPR) to validate the binding of Cepharanthine to HSP90AB1. About 8–10 μg of purified HSP90AB1 proteins were coupled to a CM5 sensor chip (GE healthcare) with an immobilization level of ~15000 RU. Different concentrations of Cepharanthine were injected on the chip, and the affinity constant (*K*_D_) was tested using SPR. RU, relative response unit. **c** Target engagement assay of Cepharanthine. CETSA showed that Cepharanthine targeted HSP90AB1. Top, western blot, HSP90AB1 protein was detected using a specific antibody after the A549 cells were treated with Cepharanthine (100 μM) or DMSO (1%, as control) and heated. Bottom, quantification of the western blot. Data are means ± SD, *n* = 3 biologically independent samples. ***P < 0.001, **P < 0.01, *P < 0.05. Two-tailed student’s *t*-test. **d** Schematic for virus infection and drug administration for mice. Drug or vehicle (2% DMSO, 30% PEG-300, and 5% Tween-80) was administered intranasally to hACE2 transgenic mice (Cepharanthine or CsA, 10 mg/kg) one day before viral challenge. The mice were subsequently subjected to intranasal challenge with SARS-CoV-2 (with 10^5^ TCID_50_, IPBCAMS-YL01/2020). Lung tissues were sampled at the indicated time points. *n* = 3 mice were euthanized at 3 dpi and 5 dpi respectively, for each drug and vehicle control group. **e** Antiviral effects of Cepharanthine and CsA against SARS-CoV-2 in vivo. As in **d**, 6 hACE2 mice were infected with SARS-CoV-2 and treated with Cepharanthine, CsA or the DMSO vehicle solution, and were sacrificed at 3 or 5 dpi. Viral loads in the lungs of these mice were quantified by qPCR. Data are shown as means ± SEM. *P < 0.05, **P < 0.01. Two-tailed student’s *t*-test. **f**, **g** The expression of pro-inflammatory cytokines in lungs of SARS-CoV-2-infected mice. As in **d**, the hACE2 transgenic mice were infected with SARS-CoV-2 and treated with Cepharanthine or CsA. The expression levels of *TNF* (**f**) and *IL6* (**g**) in mouse lung relative to those in the vehicle treatment group were quantified by qPCR at 5 dpi. For **f**, **g**, *n* = 3. Data are shown as means ± SEM, *P < 0.05, Two-tailed student’s *t*-test. **h** H&E staining of lung tissues of mice infected with SARS-CoV-2. As in **d**, upon SARS-CoV-2 infection, mice were intranasally given Cepharanthine or CsA (10 mg/kg daily). Lung tissues were collected at 3 dpi and 5 dpi for H&E staining assay. Fluid exudates and inflammations are indicated by black arrow. Scale bar, 200 μm. **i** Antiviral effects of co-treatment with CsA and Trifluoperazine (left), or Cepharanthine and Trifluoperazine (right). CsA or Cepharanthine was combined with Trifluoperazine as the indicated concentrations. Huh7.5.1 cells were treated with the combined drugs and infected with the B.1.351 strain of SARS-CoV-2 (MOI 0.05). Cells treated with vehicle without drugs (DMSO) were used as control. Viral loads in culture supernatants were quantified at 48 h post infection by qPCR (represented as percentage relative to the DMSO-treated samples). The highest single agent model^72,73^ was used to assess the pharmacological interactions (average synergy) between Trifluoperazine and CsA (left), and between Trifluoperazine and Cepharanthine (right). Data are means ± SEM. *n* = 3 biologically independent samples.

We then assayed the antiviral potency and cytotoxicity at different concentrations for the selected five drugs in the human lung cell line A549^ACE2^^46^ infected with bona fide SARS-CoV-2 virus (IPBCAMS-YL01/2020) (the first row of Fig. 5a). We found that the immunosuppression drug Cyclosporin A (CsA), which targets the positive-strand RNA virus-interacting protein Cyclophilin A (PPIA), inhibited SARS-CoV-2 (WT) infection with an IC50 (the half maximal inhibitory concentration) value of 1.52 µM and a CC50 (the half maximal cytotoxic concentration) value of > 30 μM in A549^ACE2^ (a selective index (SI) > 19.74). The heat shock protein inhibitor Cepharanthine inhibited SARS-CoV-2 (WT) infection with an IC50 value of 1.67 µM and a CC50 value of 30.92 µM (SI = 18.51). And the cytoskeleton disruptor Trifluoperazine also showed strong activity against SARS-CoV-2 (IC50 of 3.46 µM, CC50 value of 36.98 µM, SI = 10.69). The antiviral effects of CsA, Cepharanthine, and Trifluoperazine were also confirmed for SARS-CoV-2 infection in human liver Huh7.5.1 cells (the first row of Supplementary information, Fig. S5c).

Given that our vRNA interactome datasets showed interactions between both ZIKV and EBOV vRNAs and heat shock proteins (e.g., HSP90, HSPA9, etc.) as well as cytoskeleton-related proteins (e.g., TUBA1C, TUBB), we also examined the antiviral effects of Cepharanthine and Trifluoperazine with infection assays against ZIKV in Huh7 cells (MR766, MOI 0.5) and against EBOVΔVP30-GFP (MOI 0.1) in Huh7.5.1-VP30 cells^22^ (the second and third row of Fig. 5a), respectively. Trifluoperazine inhibited ZIKV infection with an IC50 value of 0.96 µM, and a CC50 value of 7.79 µM (SI = 8.11), and the most potent anti-ZIKV effect we detected was for Cepharanthine (IC50 value of 2.19 µM, CC50 value of 24 µM, SI = 10.97). Both of these agents also conferred antiviral activities against EBOVΔVP30-GFP virus infection (Trifluoperazine: IC50 = 1.79 µM, SI = 2.18; and Cepharanthine: IC50 = 0.42 µM, SI = 12.10). Collectively, these results support that the FDA-approved cytoskeleton disruption drug Trifluoperazine and the heat shock protein inhibitor Cepharanthine (approved in Japan for treating alopecia) have broad-spectrum antiviral activity against the three major pathogenic RNA viruses of our study.

### Confirming the specific involvement of the targeted host factors in the observed antiviral effects

We conducted confirmatory studies to test whether the select drugs inhibit viral infection through targeting their known target proteins. Note that our earlier results showed that knockdown of heat shock proteins (HSP90AB1, HSPA9, and HSPD1) reduced the SARS-CoV-2 vRNA levels in Huh7.5.1 cells (Fig. 4a) and our uvCLAP data confirmed the direct physical interaction between HSP90AB1 and SARS-CoV-2 vRNA (Fig. 4c), supporting that heat shock proteins in host cells can influence SARS-CoV-2 infection. We next performed Surface Plasmon Resonance (SPR) to test the affinity of Cepharanthine for HSP90AB1. These experiments used the IDH2 protein and its well-known inhibitor Enasidenib^47^ as positive controls. Affinity measurements showed that Enasidenib bound to IDH2 with dissociation constant (*K*_D_) = 0.61 µM (Supplementary information, Fig. S5d) and that Cepharanthine binds to HSP90AB1 with a *K*_D_ value of 3.5 µM (Fig. 5b).

We also performed a cellular thermal shift assay (CETSA)^48^ which showed that Cepharanthine bound to HSP90AB1 in cells, using Enasidenib binding to IDH2 as a control (Fig. 5c; Supplementary information, Fig. S5e). And our CETSA experiments showed that Trifluoperazine slightly affected the thermal stability of TUBB (Supplementary information, Fig. S5f). TUBB is a component of the cytoskeleton, and distinct from our findings for HSPs, knockdown of TUBB resulted in only minimal inhibitory effects on SARS-CoV-2 infection (Fig. 4a). Further supporting the target engagement of Trifluoperazine in Caco-2 cells, immunofluorescence with an anti-TUBB antibody showed that the architecture of the TUBB cytoskeletal networks was disrupted by Trifluoperazine (Supplementary information, Fig. S5g).

### Cepharanthine and CsA potentially inhibit SARS-CoV-2 replication in vivo

We also worked with hACE2 transgenic mice^49^ to test the in vivo antiviral effects of Cepharanthine and CsA in mice exposed to SARS-CoV-2 (Fig. 5d–h). We observed very slight weight loss of hACE2 mice challenged with SARS-CoV-2 (Supplementary information, Fig. S6a). Mice were given daily intranasal administration of 10 mg/kg Cepharanthine or CsA (or vehicle). Although drug administrations for the hACE2 mice were one day before SARS-CoV-2 infection, the viral loads of the drug-treatment groups showed no obvious differences at 3 dpi but a significant reduction at 5 dpi, compared with that of the vehicle-treatment group (Fig. 5e). We also performed a two-way ANOVA analysis to evaluate the effects of time points (3 dpi vs 5 dpi) and drug treatments (Cepharanthine- or CsA- vs vehicle-treatment) on viral loads of SARS-CoV-2 in our study, and found that the combination of time and drug treatment has significant reduction effect on viral loads, for each drug (*P* = 0.03 for Cepharanthine-treatment group and *P* = 0.002 for CsA-treatment group). Plaque assay revealed that the infective virions reduced more than 10 fold in lung tissue of CsA-treated mice (Supplementary information, Fig. S6b). Consistent with a previous report,^49^ we did not observe infective virus in other mouse organs, including the brain or intestines. The expression levels of *TNF* and of *IL6* were reduced in the Cepharanthine and CsA groups compared to the vehicle group (Fig. 5f, g). Consistent with previous findings,^50^ the vehicle group SARS-CoV-2-infected mice showed inflammation in lung tissues, which contained a protein-rich fluid exudate (Fig. 5h). We still observed some injuries and inflammation in the Cepharanthine- and CsA-treated animals, but the extent of damage was much relieved in lung tissue (Fig. 5h). As CsA and Cepharanthine are approved drugs that have been used in the clinic for decades, and their safety and pharmacokinetics have been extensively evaluated (e.g., the reported C_max_ values of CsA and Cepharanthine in human serum or mice brain ranged from 6.9 to 300 µM, which were much higher than the IC50 values observed in our study),^51–53^ our promising in vivo results warrant the continued development of Cepharanthine and CsA as potential COVID-19 therapies.

### Combination therapies confer potent antiviral activity against the recently emerged SARS-CoV-2 B.1.351 variant

Mutations occurring in viral genomes raise the risk of SARS-CoV-2 evolving to escape neutralizing antibodies and vaccines.^54–56^ Thus, by targeting host factors essential for virus infection, host-directed antiviral drug discovery is especially relevant in the context of an ongoing pandemic which features the apparently frequent emergence of mutated SARS-CoV-2 variants. Working with a SARS-CoV-2 variant (B.1.351),^57^ we tested the performance of three antiviral drugs identified in this study, as well as two FDA-approved and SARS-CoV-2-infection-suppressing drugs identified in our previous study that were predicted to target vRNA-binding host factors (Sorafenib and Deguelin).^58^ CsA, Cepharanthine, Trifluoperazine, Sorafenib, and Deguelin all exerted inhibitory effects in B.1.351-infected cells, among which CsA and Cepharanthine showed the lowest IC50 values of 0.996 µM and 0.24 µM in human lung cell line A549^ACE2^, and that were 0.73 µM and 0.06 µM in Huh7.5.1 cells, respectively (the fourth row of Fig. 5a; the second row of Supplementary information, Fig. S5c).

Finally, we used combinations of drugs which target distinct host proteins to explore potential improvements in anti-viral efficacy against SARS-CoV-2 infection. We tested combinations of the aforementioned five drugs (in pairs) against B.1.351 in assays with Huh7.5.1 cells (Fig. 5i; Supplementary information, Fig. S6c–f). Briefly, the most striking result was for a combination comprising Cepharanthine and Trifluoperazine (5 µM each), which reduced vRNA levels to less than 0.01% compared to vehicle-control group cells. Note that this inhibition level is approximately 50 fold stronger than Cepharanthine alone and 1000 fold stronger than Trifluoperazine alone (Fig. 5i, right). None of the tested combinations caused significant cytotoxicity (Supplementary information, Fig. S6e, f). These promising results support that host-directed antiviral drug discovery as an important strategy to ensure treatments against emerging SARS-CoV-2 variants.

## Discussion

Our ChIRP-MS profiling of the vRNA–host protein interactomes of human cells infected with the causal pathogenic viruses for COVID-19, Zika, and Ebola virus diseases identified interaction patterns that reflect both common and virus-specific host proteins which regulate vRNAs. These interactome datasets provide a rich resource to help illuminate the infection biology for these three pathogenic RNA viruses and potentially others as well. The RNA-centric view of ChIRP-MS is particularly informative in revealing the molecular virology processes occurring during multiple stages of the life cycle of RNA viruses in infected cells.^12^

Many host factors for SARS-CoV-2 infection have been identified by genome-wide CRISPR screenings.^59,60^ Among the 12 vRNA interactors that were identified as functional for SARS-CoV-2 infection by our shRNA screening, two (MATR3 and NONO) were also identified by the CRISPR screenings. Interestingly, IGF2BP1 and HSP90AB1, which were identified to bind vRNA and regulate infection of SARS-CoV-2 in our study, were not in the list from the CRISPR screenings,^59,60^ suggesting that our interactome-informed focused screening can serve as a complementary assay to identify more functional host factors.

Therefore, in depth mechanistic study of the vRNA interactomes may help understand how the vRNAs can hijack host proteins to support viral replication. Consider that besides our demonstration that IGF2BP1 promotes translation of vRNA, our vRNA interactome and uvCLAP data may inspire following studies for the host factor HNRNPA2B1, a protein reported as an m^6^A reader responsible for microRNA processing and alternative splicing.^61^ Recently, mutations of m^6^A modification sites within the SARS-CoV-2 genome were linked to viral infection and transmission.^62^ In SARS-CoV-2 infection, HNRNPA2B1 may function as a regulator of vRNA through its recognition of m^6^A modifications.

Comparative interactome analysis for the three examined RNA viruses offered a straightforward way to explore and visualize common and virus-specific interactions. Our analyses also enabled informed predictions about which inhibitors are likely to be virus-specific or broad-spectrum antiviral agents. Ultimately, these efforts demonstrated that agents targeting heat shock proteins (Cepharanthine) and cytoskeleton components (Trifluoperazine) can exert broad-spectrum effects against all three RNA viruses we examined in our study.

Although many studies have screened for antivirals against SARS-CoV-2 infection,^63–66^ these studies have not addressed whether the drugs target virus-interacting proteins per se. We compared the five drugs (i.e., CsA, Silvestrol, Cepharanthine, Trifluoperazine, and Enasidenib) that we identified from our interactome-informed drug repurposing study against a recently published dataset from screening of approximately 12,000 compounds for SARS-CoV-2 antivirals^66^. Only Cepharanthine is in their screening list, which showed an inhibition ratio > 40% at 2.5 μM. We also compared our identified drugs with a drug repurposing screens for virus entry inhibitors containing 2678 compounds, again, Cepharanthine in their list showed antiviral activities with IC50 value of 1.4 μM^65^. We also noticed that two other groups also validated the activity of Cepharanthine against SARS-CoV-2 very recently, with the reported IC50 value ranging from 0.4 to 4.47 μM^67,68^, as that was 1.67 μM by our experiment.

Focusing on COVID-19, the emergence of variants may render neutralizing antibodies or other virus-targeting therapies ineffective^54–56^. Our interactome-informed targeting of the host factors essential for virus infection identified drugs including CsA, Cepharanthine, and Trifluoperazine, which all exert inhibitory effects against the B.1.351 lineage, highlighting the promise of these agents for treating infections by SARS-CoV-2 variants which may have evolved to escape antibody neutralization.

Looking forward, we anticipate that deeper explorations of the antiviral modes of action for Cepharanthine, Trifluoperazine, and CsA will reveal biological insights about how pathogenic RNA viruses infect cells, how host cells respond, and which (if any) countermeasures the viruses may deploy to overcome host defense pathways. In sum, our study provides rich interactome datasets, illustrates how to profitably integrate such data to drive biological and medical discoveries, and reveals basic insights about the pathogenic and host–pathogen interaction mechanisms of three RNA viruses that have profoundly impacted human society.

## Materials and methods

### Data reporting

No statistical methods were used to predetermine sample size. The experiments were not randomized, and the investigators were not blinded to allocation during experiments or during outcome assessment.

### Cell lines and antibodies

The cell lines and antibodies used in this study are listed in Supplementary information, Table S7. All cell lines were determined to be free of mycoplasma based on PCR and nuclear staining. All cell lines mentioned in this study were cultured in DMEM (10% FBS, 1× Antibiotic-Antimycotic) at 37 °C under 5% CO_2_.

### Virus strains and cell infection

For SARS-CoV-2 infection, Huh7.5.1 cells were cultured in T-175 flasks (2 × 3 × 3 flasks, including three biological repeats, three flasks for each mock or infected ChIRP-MS experiment), at a density of 5 × 10^6^ cells. The cells were briefly washed with DMEM at 16 h after seeding, and incubated with a clinical isolate of SARS-CoV-2 (IPBCAMS-YL01/2020) for 1 h at the MOI of 0.05. Then the cells were supplemented with DMEM maintenance medium containing 1% FBS and cultured at 37 °C, 5% CO_2_ for an additional 30 h. The cultured cells were washed twice with PBS, and 4% formaldehyde (Pierce, 28908) was added for crosslinking at room temperature for 4 h. Live virus was inactivated by an additional 12 h incubation with 4% formaldehyde at 4 °C. The cells were then collected and washed with 0.125 M Glycine at room temperature, followed by centrifugation at 1000× *g*, 4 °C for 5 min and three washes with PBS. The cell pellets were used for ChIRP-MS experiments. Mock cells (no infection) were cultured and treated the same as the infected cells. All experiments involving live SARS-CoV-2 in this study were performed in a biosafety level 3 facility.

For ZIKV infection, 7 × 10^6^ Huh7 cells were cultured on a 15-cm dish for 20 h (2 × 3 × 3 plates, including three biological repeats, three plates for each mock or infected ChIRP-MS experiment), then infected with ZIKV (MR766, MOI 0.5). After 72 h, cells were collected using trypsin digestion and washed twice with cold PBS, followed by crosslinking in PBS containing 3% formaldehyde at room temperature for 30 min. Crosslinking was stopped by adding a 1/10 volume of room temperature 1.25 M Glycine for 5 min. The cells were then washed three times with PBS. After centrifugation, PBS was removed and the cell pellets were used for ChIRP-MS. Mock cells were cultured and treated like infected cells, but no virus was added.

EBOVΔVP30-GFP^22^ (strain Zaire Mayinga) virions were generated using VP30-expressing Vero E6 cell lines, then used to infect Huh7.5.1 cells expressing VP30 (Huh7.5.1-VP30) at the MOI = 0.1. For the EBOV ChIRP-MS experiment, Huh7.5.1-VP30 cells were cultured on 15-cm plates (2 × 3 × 2 plates, including two biological repeats, three plates for each mock or infected ChIRP-MS) at a density of 6 × 10^6^ cells, then infected with EBOVΔVP30-GFP virus. After 72 h, infected and mock cells were collected and crosslinked as in the ZIKV experiment.

### Identifying host proteins interacting with vRNA by ChIRP-MS

ChIRP-MS was performed according to a previous report^16^ with some modifications. Briefly, crosslinked cells (~100 mg) were resuspended in 1 mL lysis buffer (containing 50 mM Tris–HCl, pH 7.0, 10 mM EDTA, 1% SDS, 0.1% sodium deoxycholate, 0.5% DDM and 0.1% NP-40) and sonicated. Probes (2 μL of 100 μM) tiling the whole viral genome (Supplementary information, Table S1) were used to capture vRNA–protein complexes. MyOne C1 beads (Invitrogen, 65001) were blocked with 3% BSA and yeast tRNA (Solarbio, T8630), and 200 μL of C1 beads was used for each ChIRP experiment. The captured materials were first incubated twice with lysis buffer for 5 min, then washed four times with ChIRP wash buffer (2× SSC, 0.5% SDS) for 5 min. The co-purified proteins were reverse crosslinked using 0.3 M NaCl buffer (7.5 mM HEPES, pH 7.9, 12.5 mM d-biotin, 0.3 M NaCl, 1.5 mM EDTA, 0.2% SDS, 5 mM DTT, 0.075% sarkosyl, and 0.02% sodium deoxycholate) at 70 °C with shaking for 1 h. Proteins were precipitated in 20% TCA, then the protein precipitate was resuspended in RIPA buffer (25 mM Tris-HCl, pH 7.4, 1% NP-40, 0.5% DOC, 0.1% SDS). Protein samples were loaded onto an SDS-PAGE gel and visualized using silver staining. Protein bands were excised from the gel and destained following product instructions (Pierce Silver Stain for Mass Spectrometry, 24600). The excised gel bands were desalted, pH adjusted, and digested overnight using Trypsin. The digested peptides were extracted from the gel using 50% acetonitrile and 0.1% formic acid, and analyzed via mass spectrometry (Thermo Scientific Q Exactive). ChIRP-MS control experiments were performed on cells without viral infection (mock) by using the same set of probes.

To define the proteins specifically recruited by vRNA during infection, we transfected the viral RNA segments into cells; subsequently, ChIRP-MS experiments were performed using the transfected samples as the “segment transfection” control. Specifically, we cloned segments of the viral genome (12 segments, covering the full length of the SARS-CoV-2 genome, or six segments for the ZIKV genome or nine segments for the EBOV genome) into the pCDNA3 expression vector. These plasmids were then transfected into Huh7 (ZIKV) or Huh7.5.1 cells (SARS-CoV-2 and EBOV) to express the segmented vRNA. At 24 h post transfection, expressions of viral RNA segments in the transfected cells were evaluated using qPCR. The same primer pairs that target different viral RNA segments were used for detecting vRNA in the virus-infected samples. The transfected cells were then collected for “segment transfection” ChIRP-MS by using equal amounts of sample as the virus infection experiments and the same set of probes.

To determine the vRNA recovery rate for ChIRP-MS (percentage of RNA retrieve), 10 μL of lysate (1%) was removed from 1 mL of sonicated cell lysate and used as the input sample. At the last wash, a 10 μL volume of wash buffer containing beads (1%) was removed as the eluate for RNA quantification. Samples (input and eluate) were suspended in 100 μL PK buffer (10 mM Tris-HCl, pH 7.0, 100 mM NaCl, 0.5% SDS, 1 mM EDTA) containing 20 μg/mL proteinase K (Roche, 3115879001). The input and eluate samples were incubated at 50 °C for 45 min and then 95 °C for 10 min with mixing. Finally, 300 μL of TRIzol LS reagent was added to extract RNA following the manufacturer’s instructions. For both input and eluate, 2 μL of RNA was used for reverse transcription for cDNA synthesis using a PrimeScript RT reagent Kit (TAKARA, RR047A). For EBOV, sequence specific primers for the EBOV genome and *GAPDH* mRNA were used for reverse transcription. qPCR of vRNA and *GAPDH* were performed using SYBR Green kits (TAKARA, RR420A) following the manufacturer’s instructions. The following primers were used for reverse transcription or qPCR:

*GAPDH* Forward primer: ACACCCACTCCTCCACCTTTGAC;

*GAPDH* Reverse primer: ACCCTGTTGCTGTAGCCAAATTC.

ZIKV E Forward primer: CCGCTGCCCAACACAAGGTGAAG;

ZIKV E Reverse primer: CCACTAACGTTCTTTTGCAGACAT.

SARS-CoV-2 NP Forward primer: GGGGAACTTCTCCTGCTAGAAT;

SARS-CoV-2 NP Reverse primer: CAGACATTTTGCTCTCAAGCTG.

EBOV NP Forward primer: CCGTTCAACAGGGGATTGTTCG;

EBOV NP Reverse primer: CTGCTGGCAGCAATTCCTCAAG.

EBOV reverse transcription primer: CTCAGAAAATCTGGATGGCGCCGAGTCTC

*GAPDH* reverse transcription primer: CTGAGTGTGGCAGGGACTCCCCAG

### ChIRP-WB

For ChIRP-WB, Huh7.5.1 (SARS-CoV-2), Huh7.5.1-VP30 (EBOV), or Huh7 (ZIKV) cells were cultured on ten 10-cm plates, infected with virus, and crosslinked as above described. Then, ~150 mg mock or infected cells were resuspended in 1 mL lysis buffer and sonicated. ChIRP experiments were performed as described above. 10 μL of cell lysate (per 1 mL, 1%) was removed to be used for western blotting. After washing, MyOne C1 beads were resuspended using 50 µL of 0.3 M NaCl elution buffer (7.5 mM HEPES, pH 7.9, 12.5 mM d-biotin, 0.3 M NaCl, 1.5 mM EDTA, 0.2% SDS, 5 mM DTT, 0.075% sarkosyl and 0.02% sodium deoxycholate), boiled at 70 °C for 1 h, and then at 95 °C for 30 min to elute proteins. 10 μL of the protein eluate and input samples were separated on SDS-PAGE gels and immunoblotted using the specific antibodies listed in Supplementary information, Table S7.

### Define core and expanded interactomes from ChIRP-MS results

The ChIRP-MS data included three groups of experiments for each virus (SARS-CoV-2, ZIKV and EBOV), i.e., ChIRP-MS of the virus-infected samples (“virus infection”), samples without virus infection (the “mock” control), and samples from experiments with viral RNA segment transfection (the “segment transfection” control, a new type of control for excluding putative nonfunctional proteins co-precipitating with vRNA fragments). Proteomic data were filtered by applying a minimum Protein Score of 1.5. The vRNA-interacting proteins were scored with the MiST scoring algorithm^69^ using default parameters. We used the same data analysis pipeline for all samples, and calculated protein enrichment for the “virus infection” data over the “mock” data (termed as the “expanded” interactome), as well as the enrichment of the “virus infection” data over the “segment transfection” data. The shared proteins between the resulted enriched protein using the “mock” control and using the “segment transfection” control were defined as the “core” interactome.

Specifically, raw mass spectrometry data were processed with Proteome Discover using the built-in search engine to search against the human proteome (Uniprot database). Viral proteins, including SARS-CoV-2 (NC_045512.2), ZIKV (NC_012532.1), and EBOV (NC_002549.1) proteins were downloaded from NCBI and added into the database manually. For SARS-CoV-2 and EBOV infected samples, proteins identified with a MiST score > 0.6 were considered to interact with vRNA. For ZIKV-infected samples, the cutoff MiST score was set as > 0.7. Every identified protein was assessed with the same parameters as in MiST, i.e., “Abundance” to represent protein abundance in virus-infected samples, defined as the mean of the bait-prey quantities (spectral counts divided by protein length) over all replicates for virus-infected samples; “Specificity” to measure the uniqueness of proteins identified in virus-infected samples compared with noninfected samples (mock), defined as the proportion of the abundance of virus-infected samples compared to the abundance of noninfection samples; “Reproducibility” to evaluate the variance of each identified protein abundance among replicates, defined as the normalized entropy of the bait-prey quantities over all replicates for virus-infected samples. The proteins interacting with at least two vRNAs were defined as common interacting proteins.

### Protein domain analysis

To analyze whether there were possible enriched protein domains among the identified proteins, we annotated the domains of identified proteins using the Uniprot database. For each protein domain, we counted the number of identified proteins containing this domain. The enrichment (odds ratio) and significance *P* value of each domain among identified proteins, compared to all human proteins, was calculated by Fisher’s exact test.

$${{{{{{{\mathrm{odds}}}}}}}}\;{{{{{{{\mathrm{ratio}}}}}}}} = \frac{{\frac{{{{{{{\rm{number}}}}}}\;{{{{{\rm{of}}}}}}\;{{{{{\rm{identified}}}}}}\;{{{{{\rm{proteins}}}}}}\;{{{{{\rm{containing}}}}}}\;{{{{{\rm{the}}}}}}\;{{{{{\rm{domain}}}}}}}}{{{{{{{\rm{number}}}}}}\;{{{{{\rm{of}}}}}}\;{{{{{\rm{identified}}}}}}\;{{{{{\rm{proteins}}}}}}\;{{{{{\rm{not}}}}}}\;{{{{{\rm{containing}}}}}}\;{{{{{\rm{the}}}}}}\;{{{{{\rm{domain}}}}}}}}}}{{\frac{{{{{{{\rm{number}}}}}}\;{{{{{\rm{of}}}}}}\;{{{{{\rm{all}}}}}}\;{{{{{\rm{proteins}}}}}}\;{{{{{\rm{containing}}}}}}\;{{{{{\rm{the}}}}}}\;{{{{{\rm{domain}}}}}}}}{{{{{{{\rm{number}}}}}}\;{{{{{\rm{of}}}}}}\;{{{{{\rm{all}}}}}}\;{{{{{\rm{proteins}}}}}}\;{{{{{\rm{not}}}}}}\;{{{{{\rm{containing}}}}}}\;{{{{{\rm{the}}}}}}\;{{{{{\rm{domain}}}}}}}}}}$$

### Protein complex analysis

To analyze whether there were possible enriched protein complexes among the identified proteins, we performed a protein complex enrichment analysis using the CORUM database^70^. For each protein complex, we counted the number of identified proteins in this complex. The enrichment (odds ratio) and significance *P* value of these proteins in each complex, compared to all human proteins, was calculated by Fisher’s exact test.

$${{{{{{{\mathrm{odds}}}}}}}}\;{{{{{{{\mathrm{ratio}}}}}}}} = \frac{{\frac{{{{{{{\rm{number}}}}}}\;{{{{{\rm{of}}}}}}\;{{{{{\rm{identified}}}}}}\;{{{{{\rm{proteins}}}}}}\;{{{{{\rm{in}}}}}}\;{{{{{\rm{complex}}}}}}}}{{{{{{{\rm{number}}}}}}\;{{{{{\rm{of}}}}}}\;{{{{{\rm{identified}}}}}}\;{{{{{\rm{proteins}}}}}}\;{{{{{\rm{not}}}}}}\;{{{{{\rm{in}}}}}}\;{{{{{\rm{complex}}}}}}}}}}{{\frac{{{{{{{\rm{number}}}}}}\;{{{{{\rm{of}}}}}}\;{{{{{\rm{all}}}}}}\;{{{{{\rm{proteins}}}}}}\;{{{{{\rm{in}}}}}}\;{{{{{\rm{complex}}}}}}}}{{{{{{{\rm{number}}}}}}\;{{{{{\rm{of}}}}}}\;{{{{{\rm{all}}}}}}\;{{{{{\rm{proteins}}}}}}\;{{{{{\rm{not}}}}}}\;{{{{{\rm{in}}}}}}\;{{{{{\rm{complex}}}}}}}}}}$$

We searched the significantly enriched complexes (*P* value < 0.05) for each of the three vRNA interactomes. Protein complexes present in at least two of the three virus interactomes were defined as common interacting complexes. Complexes present in only a single virus interactome were defined as virus-specific interacting complexes.

### Differential protein expression analysis

To analyze differential protein expression of SARS-CoV-2 vRNA-interacting proteins (*n* = 143) across human tissues, we obtained protein abundance values in 29 human tissues from a proteomics dataset^71^. Then the abundance of interacting proteins in each type of tissue was compared to their median protein abundance among all 29 tissues. The significance *P* value of enrichment was calculated by Mann–Whitney *U* test and adjusted by FDR.

### Gene ontology (GO) and KEGG enrichment analyses

We performed GO and KEGG pathway enrichment analyses of the identified proteins using The Database for Annotation, Visualization and Integrated Discovery (DAVID) v6.8. The significance *P* values of GO terms and KEGG pathways were calculated by Fisher’s exact test and adjusted by FDR. Top 10 enriched GO terms or KEGG pathways (with FDR < 0.05) were shown.

### Development of KO cells

Guide RNAs were designed by using the CRISPR design tool (http://crispr.mit.edu). To generate IGF2BP1-KO cells, two gRNA sequences were designed and cloned into the pX459 vector. The gRNA-containing pX459 vectors were transfected into Huh7 cells using Lipofectamine 2000 (Invitrogen, 11668027) according to the manufacturer’s instructions. The transfected cells were selected with puromycin for three days; surviving cells were digested with trypsin and diluted to one cell per 200 μL DMEM medium (10% FBS). The diluted cells were plated into 96-well plates for clonal selection. Cells containing nonsense mutations were confirmed using sanger-sequencing. The KO cell lines were further confirmed at protein expression levels by western blotting using an antibody against IGF2BP1 (RN007P, MBL).

To reexpress IGF2BP1 in the IGF2BP1-KO Huh7 cells, the IGF2BP1-coding sequence was cloned into the pLVX-Puro vector, and packaged into lentivirus by co-transfecting the packaging plasmids into HEK293T cells. The packaged lentivirus was used to transfect the IGF2BP1-KO cells for generating the “IGF2BP1 reexpression cells”. The transfected cells were selected using puromycin for four days and the IGF2BP1 reexpression cells were validated by western blotting using an antibody against IGF2BP1.

### Interactome-informed drug discovery

Briefly, to identify drugs/compounds that potentially modulate the 143 human proteins within SARS-CoV-2 expanded interactome, protein uniport IDs were searched against databases including the IUPHAR/BPS Guide to Pharmacology, Drugbank, Drugcentral, and ChEMBL. Then we retrieved 5309 compounds from these databases related to 56 host factors (Supplementary information, Table S5). Specifically, seven compounds related with five interacting proteins from the IUPHAR/BPS Guide to Pharmacology; 158 compounds related with 44 interacting proteins from Drugbank; 19 compounds related with six interacting proteins from Drugcentral, and 5125 compounds predicted to be related with 35 interacting proteins from ChEMBL (Supplementary information, Table S5). Retrieved molecules were prioritized based on their FDA approval status and availability in the Center of Pharmaceutical Technology of Tsinghua University. FDA-approved drugs were prioritized for testing of potential antiviral activities; an exception here is Silvestrol, which is an eIF4A-specific inhibitor that is currently under investigation in a registered preclinical trial.

### Antiviral drug screening

Based on the SARS-CoV-2 reference sequence (Wuhan-Hu-1, NC_045512), we cloned the SARS-CoV-2 genome as five fragments. These fragments were then assembled using in vitro ligation but replacing the viral N gene with GFP, to generate the SARS-CoV-2-GFPΔN genome. Genomic RNA of SARS-CoV-2-GFPΔN and mRNA of the N gene were in vitro transcribed using an mMESSAGE mMACHINE T7 Transcription Kit (Thermo Fisher Scientific, AM1344). Synthesized vRNA was electroporated into Vero E6-N cells that were stably expressing the SARS-CoV-2 N protein generated by lentivirus transduction, to produce the P_0_ virus. After 72 h, P_0_ virus was collected. Caco-2 cells stably expressing the N protein (Caco2-N) were generated using lentivirus infection; this Caco2-N cell line can support the infection and replication of the SARS-CoV-2-GFPΔN virus.

To screen compounds with antiviral effects using SARS-CoV-2-GFPΔN virus, Caco2-N cells were cultured on 96-well plates at a density of 1 × 10^4^ cells per well. After 24 h, cells were infected with SARS-CoV-2-GFPΔN virus at the MOI of 0.05 and analyte drugs were administered at the same time as the virus. After 72 h, fluorescence activated cell sorting (FACS) was performed to quantify GFP-expressing cells. Remdesivir was used as a positive antiviral control. The infection ratios resulting from treatment with various drug concentrations were normalized to the DMSO (0.2%)- treated cells.

Compounds, known to target common interacting proteins and which showed antiviral effects against SARS-CoV-2-GFPΔN were also tested for EBOV and ZIKV infection. To assess the antiviral effects of the compounds on EBOV, Huh7.5.1-VP30 cells were cultured on 96-well plates with 1 × 10^4^ cells per well. After 20 h, drugs were added and cells were infected at the same time with EBOVΔVP30-GFP virus (strain Zaire Mayinga) at the MOI of 0.1. After 72 h, GFP-expressing cells were counted using FACS. Remdesivir was used as a positive antiviral control. The infection ratios with various drug concentrations were normalized to the DMSO (0.4%)-treated cells.

For ZIKV, Huh7 cells were cultured on 96-well plates (Corning, 3603) with 1 × 10^4^ cells per well. To test the antiviral effects of drugs, Huh7 cells were infected with ZIKV (MR766) at the MOI of 0.5 and drugs were added at the same time. At 72 h, cells were fixed using 4% PFA, and blocked using blocking buffer (1× PBS, 5% FBS, 0.3% Triton X-100). The fixed cells were then incubated with anti-flavivirus group antigen antibody (clone 4G2, MAB10216, 1:1000) in blocking buffer for 12 h at 4 °C. After washing three times with PBS, secondary antibody (goat anti-mouse IgG H&L Alexa Fluor 488, ab150113) was diluted in blocking buffer (1:1000) containing DAPI (5 μg/mL) and was added and incubated for 2 h at room temperature. The ZIKV infection ratio was then quantified using an Opera Phenix High-content System. Total cell numbers were quantified based on DAPI staining of nuclei, and infected cells were quantified according to Alexa Fluor 488 staining in cytoplasm. The infection ratio was calculated as the number of Alexa Fluor 488-stained cells divided by the number of DAPI-stained nuclei. The infection ratios with various drug concentrations were normalized to the DMSO (0.4%)-treated cells.

For cell viability assays, Caco2-N, Huh7.5.1-VP30, and Huh7 cells were seeded on 96-well plates with 1 × 10^4^ cells per well, and treated using drugs with different concentrations. At 72 h, cell viability was measured using CellTiter-Glo Luminescent Cell Viability Assay kits (Promega, G7570). Cell viability values were normalized to DMSO-treated cells (0.2% for Caco2-N, 0.4% for Huh7 and Huh7.5.1-VP30 cells).

To screen compounds with antiviral effects using bona fide SARS-CoV-2 and the B.1.351 variant, human A549^ACE2^ and Huh7.5.1 cells (1.2 × 10^4^ cells per well) were cultured on 96-well plates. Cells were infected with SARS-CoV-2 virus (MOI 0.05, IPBCAMS-YL01/2020 or B.1.351) and analyte drugs were administered at the same time as the virus infection. At 48 h post infection, supernatants of the cultures were collected and vRNA in the supernatant was quantified by qPCR. Cells treated by different concentrations of drug but without SARS-CoV-2 infection were also cultured for 48 h and used for the cell viability assays. The vRNA and cell viability with various drug concentrations were quantified and normalized to the DMSO (0.3%)-treated cells.

Viral infection curves and cell viability curves were fitted using GraphPad Prism 8 (Nonlinear regression, Dose-response-Inhibition); IC50 and CC50 values were calculated. The SI was also calculated as the ratio of CC50/IC50.

### Drug target engagement assay

The binding of chemicals to proteins were detected by using Surface Plasmon Resonance Spectroscopy (SPR, Biacore 8 K system). 6× His-tagged HSP90AB1 and IDH2 proteins were expressed in HEK293T cells and purified by using a MagneHis Protein Purification System kit following the manufacturer’s instructions. The eluted proteins were dialyzed to change the elution buffer into coupling buffer (25 mM Tris-HCl, pH 7.5, 150 mM NaCl, 0.1% NP-40, and 10% glycerol). About 8–10 μg of purified proteins were coupled to a CM5 sensor chip (GE healthcare) following the manufacturer’s instructions with an immobilization level of ~15000 RU. Cepharanthine and Enasidenib were diluted into PBS-P supplemented with 5% DMSO and exposed to the coupled CM5 chip with the running buffer (PBS-P, 5% DMSO). Relative response level data were fitted using a saturation function (two sites, specific binding).

The CETSA was performed as previously described^48^. A549 cells were cultured on 10 cm dish, Cepharanthine or Enasidenib stock in DMSO was added to a final concentration of 100 μM and 1% DMSO concentration. Control cells were treated with DMSO of 1% final concentration. After 4 h, cells were harvested and washed twice with cold PBS. The harvested cells were resuspended with 500 μL of PBS, divided into equal aliquots (50 µL) and centrifuged at 3000× *g* for 3 min. Following removal of PBS, the aliquot cells were incubated separately at different temperatures for 3 min. Then, 50 µL of PBS was added into each tube, and cells were lysed with three cycles of freeze-thawing with liquid nitrogen. Lysates were centrifuged at 20,000 × *g* for 20 min at 4 °C. The soluble fractions were isolated for western blot analysis.

### Identifying functional vRNA-interacting proteins

shRNA-expressing plasmid vectors were purchased from the shRNA library platform of Tsinghua University. The shRNA-coding sequences were validated by Sanger-sequencing. The shRNA-expressing plasmids were packaged as lentiviruses by co-transfecting the packaging plasmids into HEK293T cells. Stable knockdown cell lines of Caco-2 or Huh7 cells were developed by lentivirus transfection following puromycin selection. The knockdown efficiencies were assessed by qPCR, using *GAPDH* as a reference. The cell lines expressing nontargeted (scramble) shRNA (NC) were used as control.

The puromycin selected gene-knockdown Caco-2 or Huh7 cells were cultured in 24-well plates and infected with SARS-CoV-2 (MOI 0.05) or ZIKV (MR766, MOI 0.5) respectively. After 48 h of infection, 20 µL supernatant of cell culture was removed and used to obtain vRNA. Then the remaining cell culture was discarded and the cells were washed twice using PBS; 1 mL of TRIzol was added for cellular total RNA extraction. Taqman probe qPCR was used to assess vRNA levels in the cell culture supernatant. To measure the intracellular vRNA levels in ZIKV-infected cells, equal quantity of total RNAs (100 ng) were reverse transcribed using the PrimeScript RT reagent Kit (TaKaRa, RR047A), then the viral RNA loads were measured by qPCR in different knockdown cells and were normalized to the levels in NC cells (expressing nontargeted shRNA).

### Immunofluorescence assay of the TUBB-containing cytoskeleton

Caco-2 cells were seeded in 12-well plates (supplemented with microslides) at 70% confluence and cultured for 12 h. Trifluoperazine in DMSO stock (0.5 mM) was added to a final concentration of 0.5 µM and 0.1% DMSO. Control cells were treated with DMSO (1% final concentration). After incubation for 6 h, the medium was removed. Then microslides were washed twice with cold PBS and fixed with 4% paraformaldehyde. After washing with PBS, slides were blocked with 1% FBS supplemented with 0.3% Triton-X100. Anti-TUBB antibody (Proteintech, 66240-1-Ig) was diluted into blocking buffer (1:500) and incubated overnight with the fixed cells at 4 °C. After washing three times with cold PBS, the slides were incubated with goat anti-mouse IgG Alexa Fluor 488 in blocking buffer (1:1000) for 1 h. The nucleus was stained using DAPI. Fluorescence was monitored by confocal microscopy (Nikon Ti-E) using the same parameter settings. The figures were then analyzed and exported using NIS-Elements software (v4.30).

### Ultraviolet crosslinking and affinity purification experiment to identify protein binding sites on vRNA

To identify the binding sites of IGF2BP1 and HSP90AB1 on vRNA, we performed a CLIP-like method known as uvCLAP^39^, with modifications. Caco2-N cells were cultured on 10-cm dishes and infected with SARS-CoV-2-GFP∆N virus with the MOI 0.05 for 48 h. Then cells were washed three times using cold PBS and crosslinked using UV254. The crosslinked cells were resuspended using 1 mL lysis buffer (50 mM Tris-HCl, pH 7.4, 100 mM NaCl, 1% Igepal CA-630, 0.1% SDS and 0.5% sodium deoxycholate) supplemented with protease inhibitor. Cells were lysed on ice for 30 min. To fragment the RNA, 10 μL of RNase I dilution (1:1000 in lysis buffer) and 2 μL Turbo DNase were added, then the cell lysates were incubated for 3 min at 37 °C with shaking at 1100 rpm. The digested lysate was then immediately transferred to ice for 3 min, followed by centrifugation of lysates at 18,000 RCF for 10 min at 4 °C. Anti-IGF2BP1 antibody (10 μg, MBL, RN007P) or anti-HSP90AB1 antibody (10 μg, Proteintech, 11405-1-AP) was conjugated to 100 μL protein A/G beads (Pierce, 88802). Rabbit IgG (10 μg) was also conjugated to protein A/G beads used for protein pull-down control. The conjugated beads were added to lysates and rotated for 1 h at 4 °C. Beads were washed twice with lysis buffer and four times with wash buffer (20 mM Tris-HCl, pH 7.4, 10 mM MgCl_2_, 0.2% Tween-20); all washes lasted 5 min with rotation at 4 °C. Co-purified RNA was dephosphorylated using T4 polynucleotide kinase (NEB, M0201L) following the manufacturer’s instructions. The beads were washed twice with PNK wash buffer, following once with high-salt wash buffer (50 mM Tris-HCl, pH 7.4, 1 M NaCl, 1 mM EDTA, 1% Igepal CA-630, 0.1% SDS and 0.5% sodium deoxycholate). Discard the high-salt wash buffer, beads were resuspended using PK buffer (100 mM Tris-HCl, pH 7.4, 50 mM NaCl, 10 mM EDTA) supplemented with 10 μL of proteinase K (20 μg/μL stock) then incubated at 37 °C for 20 min with shaking at 1100 rpm. TRIzol LS reagent (800 μL, Invitrogen, 10296028) was added to the tubes for RNA extraction as the manufacturer’s instructions. The sequencing library of IGF2BP1 or HSP90AB1 uvCLAP was prepared as the protocol described in the SMARTer smRNA-Seq Kit for Illumina User Manual.

For the sequencing data, the 1st reads were used for further analysis. The 5′- and 3′-adapter sequences were removed using Trimmomaitc (v0.30). Sequencing data were de-duplicated and the cleaned reads were then mapped to the human rRNA index with bowtie2 (v2.2.5). The unmapped reads were then mapped to the human and the SARS-CoV-2 genome with bowtie2. Peak calling was conducted using Piranha (v1.2.1) and the data tracks were produced using IGV (Integrative Genomics Viewer, v2.4.14).

### In vitro RAN stability and translation assay

The open reading frame of *IGF2BP1* was fused with a 6× His tag and cloned into the pLVX-Puro vector. The His-tagged IGF2BP1 protein was expressed in HEK293T cells and purified by using a MagneHis Protein Purification System kit following the manufacturer’s instructions. The eluted proteins were dialyzed to change the elution buffer into stock buffer (20 mM Tris-HCl, pH 7.5, 75 mM NaCl, 1 mM dithiothreitol and 5% glycerol). The purified protein was quantified using the BCA method and confirmed by SDS-PAGE with Coomassie staining. The purified IGF2BP1 protein was stored at −80 °C.

The S protein-coding sequences and UTRs of SARS-CoV-2 were cloned from virus-infected cells. Then the 5′UTR (with T7 promotor using PCR method) and 3′UTR (containing polyadenylation) were fused with the S protein-coding sequence using overlap-PCR. The cloned 5′UTR-S-3′UTR construct was validated by Sanger-sequencing, and capped RNA was generated using a HiScribe T7 High Yield RNA Synthesis Kit (NEB, E2040S) using the T7 promotor. For the cellular RNA stability and translation assay, capped 5′UTR-S-3′UTR RNA was transfected into Huh7 WT cells, IGF2BP1-KO cells, and KO cells reexpressing IGF2BP1 (KO + IGF2BP1) using Lipofectamine 2000 following the manufacturer’s instructions, followed by 4-h incubation. Then transfected cells at the indicated time points were collected for qPCR or western blotting to measure the RNA or S protein levels, respectively. For the in vitro translation assay, capped 5′UTR-S-3′UTR RNA was subjected to the retic lysate IVT system (Invitrogen, AM1200) together with the purified 6× His-IGF2BP1 protein or an equal volume of protein stock buffer. Then the in vitro translation reaction was performed following the manual. The translation of the Spike protein was detected using western blotting.

For the stability assay of the ZIKV genome, RNA of a ZIKV strain GZ01 with *RdRp* mutation (GAA) was transfected into Huh7 WT and IGF2BP1-KO cells. Four hours post transfection, cells were collected for qPCR analysis of 0 h, 12 h, 24 h, 48 h, and 72 h time points. The vRNA levels were quantified and normalized to those at the 0 h time point.

The 5′UTR and 3′UTR of MR766 were fused to the N- and C-terminal regions of Rluc, respectively, using overlap PCR to generate the 5R3 construct. The T7 promotor was added to the 5R3 and the Firefly luciferase (Fluc) constructs, respectively. The capped 5R3 and Fluc RNAs were generated using a HiScribe T7 High Yield RNA Synthesis Kit using the T7 promotor. Capped RNA (5R3) combined with Fluc RNA (molar ratio 4:1) were subjected to the retic lysate IVT system (Invitrogen, AM1200) together with the purified IGF2BP1 protein or an equal volume of protein buffer. Then an in vitro translation reaction was performed as per the manual instructions. Luciferase activities were detected using a Dual-Luciferase reporter assay kit (Promega, E1910).

### Mice and ethics statement

The hACE2-KI mice (expressing the hACE2 gene) were purchased from Jiangsu Gempharmatech, China. Mice were housed at the Institute of Laboratory Animal Science, Chinese Academy of Medical Sciences and Peking Union Medical College (CAMS and PUMC). All animal experiments were approved by the Institutional Animal Care and Use Committee of the Institute of Laboratory Animal Science, CAMS and PUMC (approval number: BYS20016) and carried out in an animal biosafety level three facility.

### Mouse challenge experiment

The in vivo antiviral effects of Cepharanthine and CsA were detected using human hACE2 transgenic mice^49^ infected with SARS-CoV-2. Cepharanthine and CsA are insoluble in water. We first dissolved Cepharanthine or CsA in DMSO to make a 100 mg/mL stock solution. We then diluted the stock using the DMSO vehicle (2% DMSO, 30% PEG-300 and 5% Tween-80). For the mouse challenge experiment, ~75 µL of drug solution or DMSO vehicle was administered intranasally to hACE2 transgenic mice (Cepharanthine or CsA, 10 mg/kg; DMSO vehicle was used as drug treatment control). Briefly, 6–8-week-old hACE2 transgenic mice (C57BL/6 background) were intranasally administered Cepharanthine or CsA (10 mg/kg) or vehicle 24 h prior to intranasal challenge with the SARS-CoV-2 virus (10^5^ TCID_50_). The drug or vehicle administration was continued once daily until two dpi. Mice were euthanized in each group at 3 dpi and 5 dpi. The lung, brain and intestinal tissues of mice were collected for viral load assays and for examinations of histopathological changes.

## Supplementary information

Supplementary information, Fig S1

Supplementary information, Fig S2

Supplementary information, Fig S3

Supplementary information, Fig S4

Supplementary information, Fig S5

Supplementary information, Fig S6

Supplementary information, TableS1

Supplementary information, TableS2

Supplementary information, TableS3

Supplementary information, TableS4

Supplementary information, TableS5

Supplementary information, TableS6

Supplementary information, TableS7

## Data Availability

The RNA sequencing data for IGF2BP1 and HSP90AB1 uvCLAP in this study are available at Gene Expression Omnibus with accession number GSE181866.
