# Supplementary material for: Comparison of viral RNA–host protein interactomes across pathogenic RNA viruses informs rapid antiviral drug discovery for SARS-CoV-2
Source: Cell Res. 2021 Nov 4;32(1):9–23. doi: 10.1038/s41422-021-00581-y (PMC8566969; doi:10.1038/s41422-021-00581-y)
Supplement: Supplementary file 1 — Supplementary information, Fig S1 [file 41422_2021_581_MOESM1_ESM.pdf]

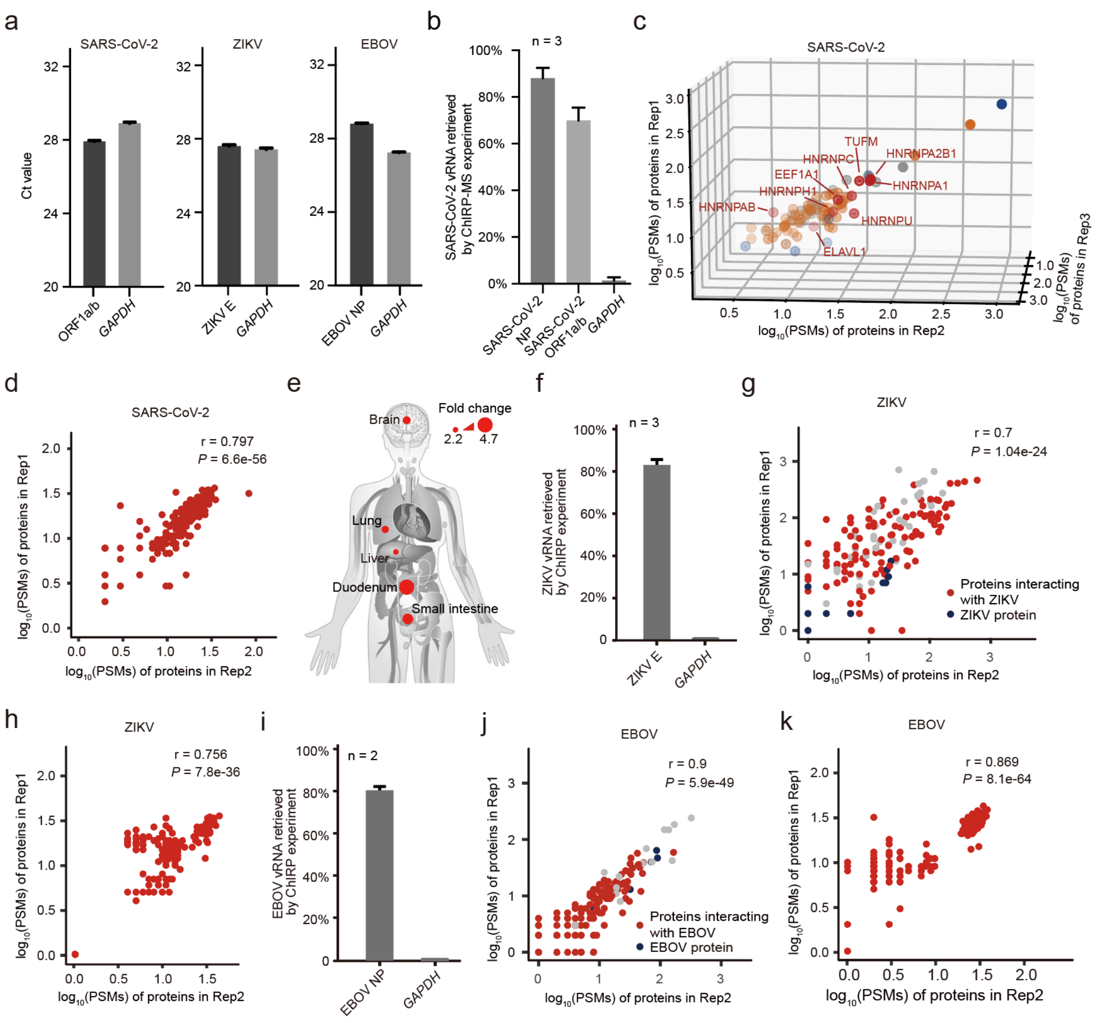

Supplementary information, Fig. S1. Identification of the human proteins interacting with SARS-CoV-2, ZIKV, and EBOV vRNA in infected cells.

a Evaluation of the vRNA levels in the infected cells that used for ChIRP-MS. The genomic RNA levels of SARS-CoV-2 (ORF1a/b, left), ZIKV (ZIKV E, middle), and EBOV (EBOV NP, right) were quantified by qPCR. The Ct values of vRNA and *GAPDH* were shown for the qPCR using the SARS-CoV-2 infected Huh7.5.1, ZIKV infected Huh7, and EBOVΔVP30-GFP infected Huh7.5.1-VP30 cells, respectively.

b, **f**, **i** The vRNA recovery rates of ChIRP-MS experiments for SARS-CoV-2 (**b**), ZIKV (**f**), and EBOV (**i**). qPCR of vRNA (SARS-CoV-2 NP and ORF1a/b gene, ZIKV E protein gene, and EBOV NP protein gene) was performed in ChIRP input and elution samples, *GAPDH* was used as a vRNA probe non-targeting control. Data are means ± SD, n = 3 or 2 biologically independent samples of ChIRP-MS as indicated.

c Correlation of $\log_{10}$ (PSMs) values for proteins from three biological replicates. Interacting proteins identified using the "mock" control are indicated as red dots (these are previously shown to interact with other coronavirus genome RNA), otherwise as orange dots; SARS-CoV-2 viral proteins co-purified with vRNA are indicated as blue dots.

d Correlation of $\log_{10}$ (PSMs) for proteins identified in cells transfected with SARS-CoV-2 RNA segments (segment transfection). Data present the Pearson correlation coefficients of $\log_{10}$ (PSMs) values from biological replicates.

e, Expression of SARS-CoV-2 RNA associating proteins in human tissues. The expanded SARS-CoV-2 vRNA interactome was intersected with a proteomic dataset of protein abundance measurement in 29 human tissues [1]. Human tissues or organs with significantly higher expression levels for SARS-CoV-2 vRNA interacting proteins (n = 143) are shown. Enrichment P value was calculated by Mann–Whitney U test and adjusted by FDR. The 5 tissues with the highest expression levels (fold change compared to median levels across all 29 tissues) of vRNA interacting proteins are shown.

g, **h**, **j**, **k** Correlation of $\log_{10}$ (PSMs) values for proteins identified in ZIKV infected Huh7 cells (**g**) or in EBOVΔVP30-GFP infected Huh7.5.1-VP30 cells (**j**) using mock as control or in the segment transfection experiments for ZIKV (**h**) or EBOV (**k**), respectively. Data present Pearson correlation coefficients of $\log_{10}$ (PSMs) values from biological replicates. The vRNA interacting proteins are indicated using red dots; viral proteins co-purified with vRNA are in blue.

Reference:

1. Wang D. et al. A deep proteome and transcriptome abundance atlas of 29 healthy human tissues. *Mol. Syst. Biol.* **15**, e8503 (2019).
