# Supplementary material for: Comparison of viral RNA–host protein interactomes across pathogenic RNA viruses informs rapid antiviral drug discovery for SARS-CoV-2
Source: Cell Res. 2021 Nov 4;32(1):9–23. doi: 10.1038/s41422-021-00581-y (PMC8566969; doi:10.1038/s41422-021-00581-y)
Supplement: Supplementary file 2 — Supplementary information, Fig S2 [file 41422_2021_581_MOESM2_ESM.pdf]

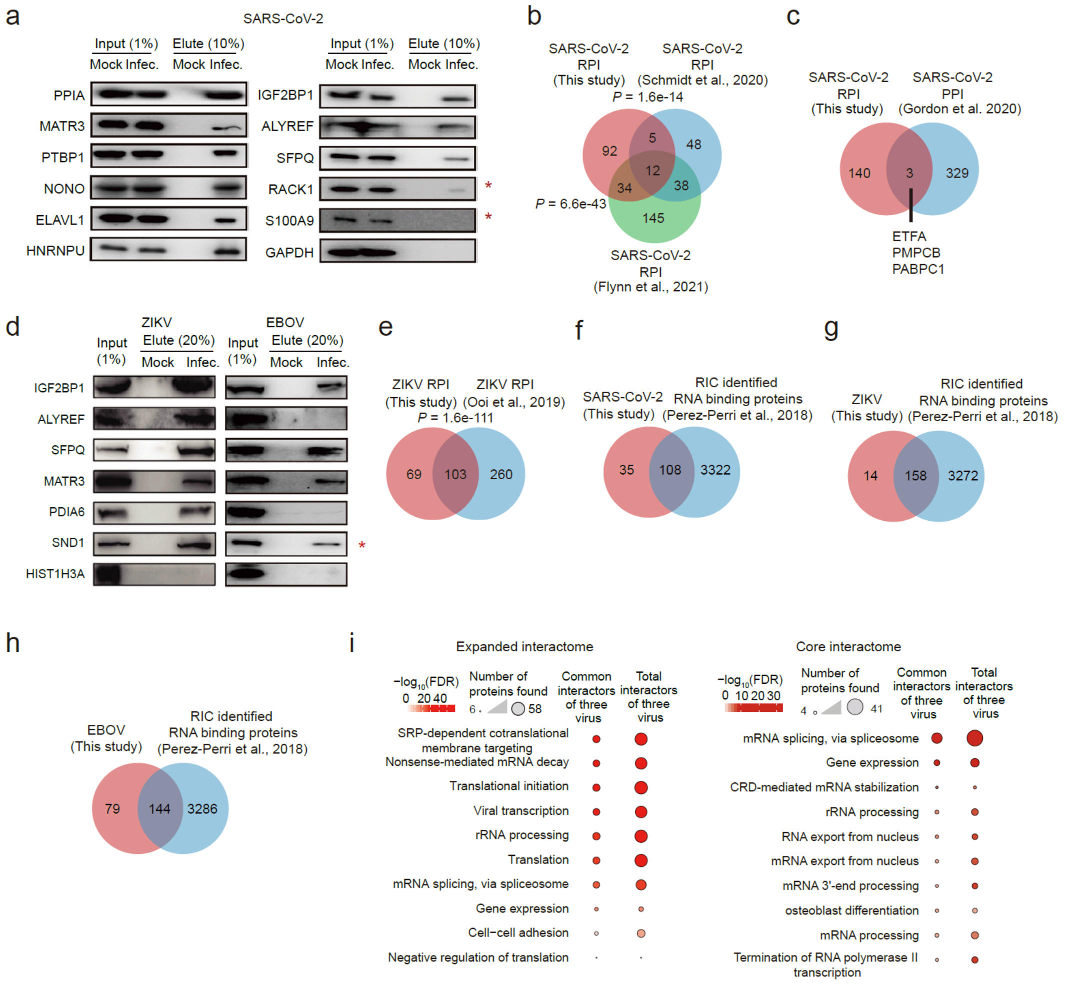

Supplementary information, Fig. S2. Confirmation and comparison of vRNA-host protein interactions.

a Validation of the interactions between the SARS-CoV-2 vRNA and human proteins using ChIRP-Western blotting (ChIRP-WB). GAPDH protein, which is not known to bind SARS-CoV-2 vRNA, was used as a negative control for ChIRP-WB experiments. Mock, cells without infection. Infec., cells infected with SARS-CoV-2. * indicates proteins that are not consistent between ChIRP-MS and ChIRP-WB.

b Comparison of SARS-CoV-2 vRNA interacting proteins identified in this study with protein groups from datasets obtained using two different RNA pull-down methods [2, 3] (Fisher's exact test).

c Comparison of SARS-CoV-2 vRNA interacting proteins with viral protein interacting proteins [4]. For **b**, **c**, RPI, vRNA-host protein interactome. PPI, viral protein-host protein interactome.

d Validation of interactions between the indicated proteins with ZIKV or EBOV vRNA using ChIRP-WB. HIST1H3A was used as a negative control. Mock, cells without infection. ZIKV or EBOV, cells infected with the indicated virus. * indicates proteins that are not consistent between ChIRP-MS and ChIRP-WB.

e ZIKV vRNA interacting proteins identified by different RNA pull-down methods. Human protein groups identified in the present study were compared with the protein groups identified by other studies [5].

f, **g**, **h** About 76% of SARS-CoV-2 (**f**), 92% of ZIKV (**g**), and 65% of EBOV (**h**) interacting proteins can directly bind RNAs. The vRNA interacting proteins identified in this study were intersected with the RNA binding proteins identified by RNA interactome capture (RIC) technology in various human cell lines [6,7].

i Gene Ontology enrichment analysis (GO) of the common and total interactors of three viruses in expanded (left) or core interactomes (right) for SARS-CoV-2, ZIKV, and EBOV, see also **Fig. 2g** (bottom). The top 10 enriched terms are shown (FDR < 0.05).

Reference:

2. Schmidt N. et al. The SARS-CoV-2 RNA-protein interactome in infected human cells. *Nat. Microbiol.* **6**, 339-353 (2021).
3. Flynn R. A. et al. Discovery and functional interrogation of SARS-CoV-2 RNA-host protein interactions. *Cell* **184**, 2394-2411 e2316 (2021).
4. Gordon D. E. et al. A SARS-CoV-2 protein interaction map reveals targets for drug repurposing. *Nature* **583**, 459-468 (2020).
5. Ooi Y. S. et al. An RNA-centric dissection of host complexes controlling flavivirus infection. *Nat. Microbiol.* **4**, 2369-2382 (2019).
6. Castello A. et al. System-wide identification of RNA-binding proteins by interactome capture. *Nat. Protoc.* **8**, 491-500 (2013).
7. Perez-Perri J. I. et al. Discovery of RNA-binding proteins and characterization of their dynamic responses by enhanced RNA interactome capture. *Nat. Commun.* **9**, 4408 (2018).
