# Supplementary material for: Comparison of viral RNA–host protein interactomes across pathogenic RNA viruses informs rapid antiviral drug discovery for SARS-CoV-2
Source: Cell Res. 2021 Nov 4;32(1):9–23. doi: 10.1038/s41422-021-00581-y (PMC8566969; doi:10.1038/s41422-021-00581-y)
Supplement: Supplementary file 3 — Supplementary information, Fig S3 [file 41422_2021_581_MOESM3_ESM.pdf]

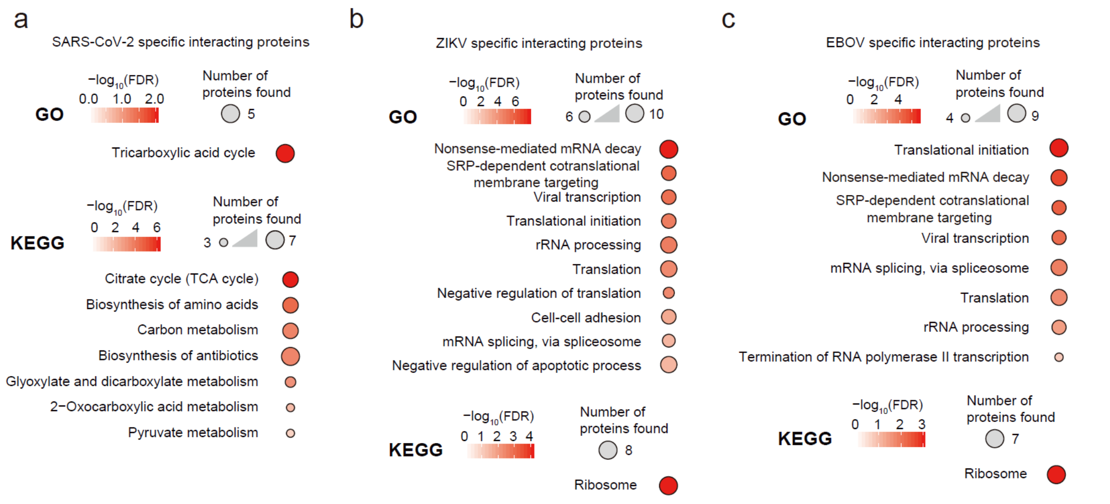

Supplementary information, Fig. S3. Functional analysis of the vRNA-specific interacting proteins.

a, **b**, **c** Gene Ontology (**GO**) and Kyoto Encyclopedia of Genes and Genomes (**KEGG**) pathway enrichment analysis of proteins specifically interacting with the SARS-CoV-2 (**a**), ZIKV (**b**) and EBOV (**c**) vRNA. For **a**–**c**, vRNA specific interacting proteins in the core interactomes of the three viruses were analyzed. The top-ranking significantly enriched terms are shown (FDR < 0.05).
