# Supplementary material for: Comparison of viral RNA–host protein interactomes across pathogenic RNA viruses informs rapid antiviral drug discovery for SARS-CoV-2
Source: Cell Res. 2021 Nov 4;32(1):9–23. doi: 10.1038/s41422-021-00581-y (PMC8566969; doi:10.1038/s41422-021-00581-y)
Supplement: Supplementary file 4 — Supplementary information, Fig S4 [file 41422_2021_581_MOESM4_ESM.pdf]

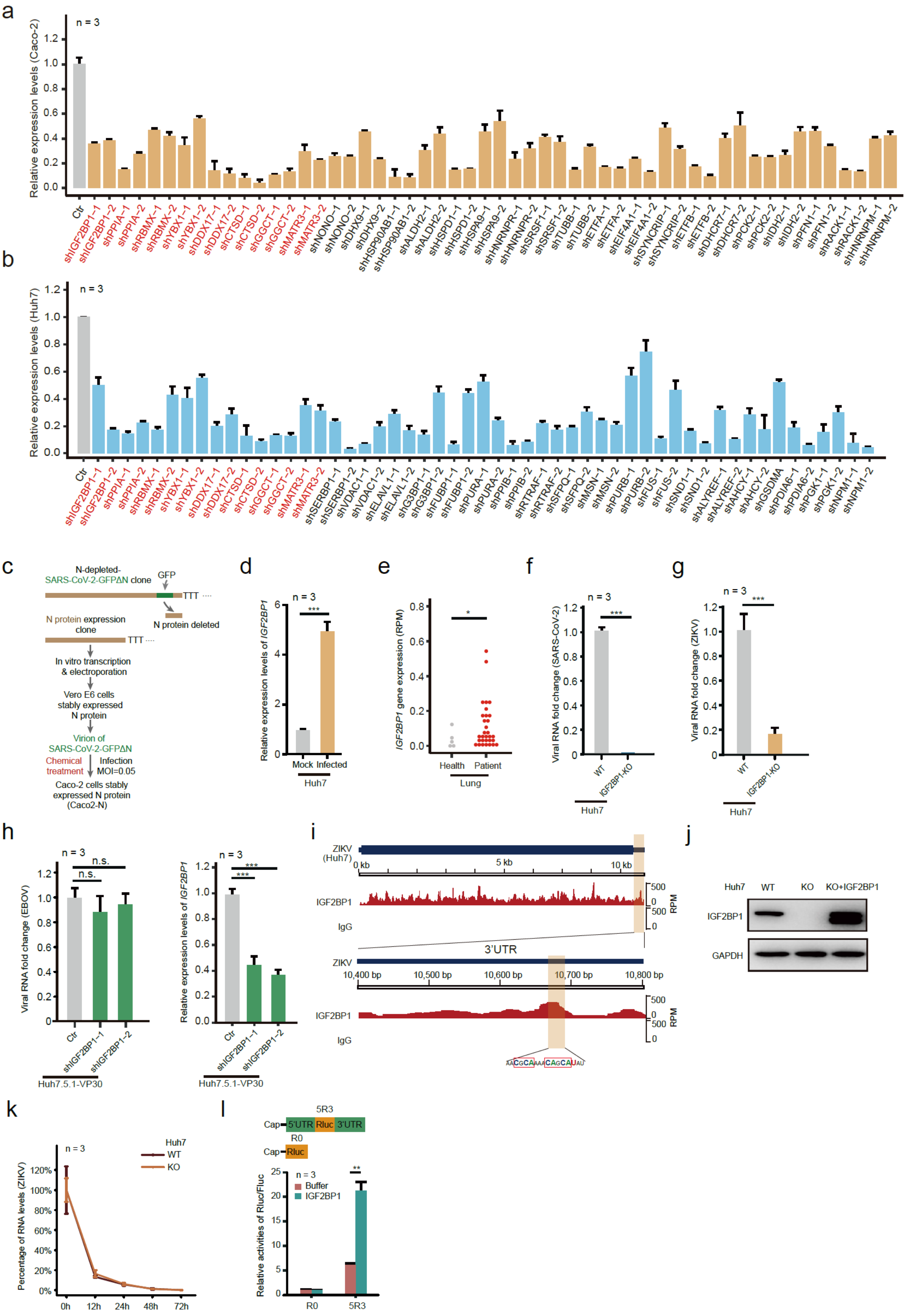

a
n = 3
Relative expression levels (Caco-2)
Ctr
shIGF2BP1-1
shIGF2BP1-2
shPPIA-1
shPPIA-2
shRBMX-1
shRBMX-2
shYBX1-1
shYBX1-2
shDDX17-1
shDDX17-2
shCTSD-1
shCTSD-2
shGGCT-1
shGGCT-2
shMATR3-1
shMATR3-2
shNONO-1
shNONO-2
shDHX9-1
shDHX9-2
shHSP90AB1-1
shHSP90AB1-2
shALDH2-1
shALDH2-2
shHSPD1-1
shHSPD1-2
shHSPA9-1
shHSPA9-2
shHNRNPR-1
shHNRNPR-2
shSRSF1-1
shSRSF1-2
shTUBB-1
shTUBB-2
shETFA-1
shETFA-2
shEIF4A1-1
shEIF4A1-2
shSYNCRIP-1
shSYNCRIP-2
shETFB-1
shETFB-2
shDHCR7-1
shDHCR7-2
shPCK2-1
shPCK2-2
shIDH2-1
shIDH2-2
shPFN1-1
shPFN1-2
shRACK1-1
shRACK1-2
shHNRNPM-1
shHNRNPM-2
b
n = 3
Relative expression levels (Huh7)
Ctr
shIGF2BP1-1
shIGF2BP1-2
shPPIA-1
shPPIA-2
shRBMX-1
shRBMX-2
shYBX1-1
shYBX1-2
shDDX17-1
shDDX17-2
shCTSD-1
shCTSD-2
shGGCT-1
shGGCT-2
shMATR3-1
shMATR3-2
shSERBP1-1
shSERBP1-2
shVDAC1-1
shVDAC1-2
shELAVL1-1
shELAVL1-2
shG3BP1-1
shG3BP1-2
shFUBP1-1
shFUBP1-2
shPURA-1
shPURA-2
shPPIB-1
shPPIB-2
shRTRAF-1
shRTRAF-2
shSFPQ-1
shSFPQ-2
shMSN-1
shMSN-2
shPURB-1
shPURB-2
shFUS-1
shFUS-2
shSND1-1
shSND1-2
shALYREF-1
shALYREF-2
shAHCY-1
shAHCY-2
shGSDMA
shPDIA6-1
shPDIA6-2
shPGK1-1
shPGK1-2
shNPM1-1
shNPM1-2
c
N-depleted-
SARS-CoV-2-GFPΔN clone
GFP
TTT
N protein deleted
N protein expression
clone
TTT
In vitro transcription
& electroporation
Vero E6 cells
stably expressed
N protein
Virion of
SARS-CoV-2-GFPΔN
Chemical
treatment
Infection
MOI=0.05
Caco-2 cells stably
expressed N protein
(Caco2-N)
d
n = 3
***
Relative expression levels of IGF2BP1
Mock
Infected
Huh7
e
*
IGF2BP1 gene expression (RPM)
Health
Patient
Lung
f
n = 3
***
Viral RNA fold change (SARS-CoV-2)
WT
IGF2BP1-KO
Huh7
g
n = 3
***
Viral RNA fold change (ZIKV)
WT
IGF2BP1-KO
Huh7
h
n = 3
n.s.
n.s.
Viral RNA fold change (EBOV)
Ctr
shIGF2BP1-1
shIGF2BP1-2
Huh7.5.1-VP30
n = 3
***
***
Relative expression levels of IGF2BP1
Ctr
shIGF2BP1-1
shIGF2BP1-2
Huh7.5.1-VP30
i
ZIKV
(Huh7)
0 kb
5 kb
10 kb
IGF2BP1
IgG
500
0
500
0
RPM
3'UTR
ZIKV
10,400 bp
10,500 bp
10,600 bp
10,700 bp
10,800 bp
IGF2BP1
IgG
500
0
500
0
RPM
j
Huh7
WT
KO
KO+IGF2BP1
IGF2BP1
GAPDH
k
n = 3
Huh7
WT
KO
Percentage of RNA levels (ZIKV)
120%
100%
80%
60%
40%
20%
0%
0h
12h
24h
48h
72h
l
5R3
Cap
5'UTR
Rluc
3'UTR
R0
Cap
Rluc
n = 3
Buffer
IGF2BP1
**
Relative activities of Rluc/Fluc
R0
5R3

Supplementary information, Fig. S4 Functional validation of vRNA interacting proteins.

a, **b** The expression levels of the indicated genes in gene-knock-down Caco-2 (**a**) and Huh7 cells (**b**). Gene expression levels were quantified in the indicated cells by qPCR relative to *GAPDH* and normalized to that in negative-control (Ctr) cells of non-targeting (scramble) shRNAs. Data are means ± SD, $n = 3$ biologically independent samples.

c Schematic diagram of the SARS-CoV-2-GFPΔN trans-complementation system. For this system, the SARS-CoV-2 genome sequence segment encoding nucleocapsid (N) was replaced by GFP, thus the resulting SARS-CoV-2-GFPΔN can only be replicated in N protein-expressing Caco-2 (Caco2-N) cells. This system can also be used for initial testing of antiviral activities; i.e., drugs or chemicals were added at the same time as the infection (MOI 0.05), and the infection ratio was then evaluated by quantifying the GFP-positive cells at 72 h post infection.

d Expression levels of *IGF2BP1* in SARS-CoV-2 infected Huh7 cells. Huh7 cells were infected with SARS-CoV-2 (MOI 0.05) for 48 h. *IGF2BP1* mRNA level was quantified relative to *GAPDH* by qPCR and normalized to that in mock cells. Data are means ± SD, $n = 3$ independent biological samples. *** $P < 0.001$. Two-tailed student's *t*-test.

e Expression levels of *IGF2BP1* in lung tissue of patients with COVID-19. The expression levels of *IGF2BP1* were analyzed using data set from a single cell sequencing experiment of COVID-19 patients and healthy people [8]. * $P < 0.05$. Two-tailed student's *t*-test.

f SARS-CoV-2 replication in IGF2BP1 knockout cells. Huh7 wild type and IGF2BP1 knockout cells were infected with SARS-CoV-2 with MOI 0.05 for 48 h. Intracellular SARS-CoV-2 vRNA level was quantified relative to *GAPDH* by qPCR and normalized to that in wild type Huh7 cells.

g ZIKV replication in IGF2BP1 knockout cells. Huh7 wild type and IGF2BP1 knockout cells were infected with MR766 (MOI 0.5) for 48 h. Intracellular ZIKV vRNA level was quantified relative to *GAPDH* by qPCR and normalized to that in wild type Huh7 cells.

h IGF2BP1 knockdown has no effects on the replication of EBOVΔVP30-GFP virus in Huh7.5.1 cells with VP30 expression. IGF2BP1 was knocked down in Huh7.5.1-VP30 cells using two distinct shRNAs (right). The gene-knock-down and control (NC, scramble non-target shRNA) cells were infected with EBOVΔVP30-GFP virus with MOI 0.1 for 48h. Intracellular EBOV vRNA level was quantified relative to *GAPDH* and normalized to that in NC cells (left). For **g–h**, data are means ± SD, $n = 3$ independent biological samples. *n.s.* not significant. *** $P < 0.001$. Two-tailed student's *t*-test.

i IGF2BP1 uvCLAP sequencing data aligned to ZIKV genome. A zoomed-in view of the specific binding site on the 3'UTR of ZIKV is shown below. The known IGF2BP1 binding motifs "CACA" are highlighted and indicated using red rectangles. IGF2BP1, sequencing data of IGF2BP1 uvCLAP. IgG, uvCLAP sequencing data of the IgG pull-down.

j Western blot to detect the expression of IGF2BP1 protein in wild type (WT), IGF2BP1 knockout (KO), and KO cells with IGF2BP1 re-expression (KO+IGF2BP1). GAPDH was used as a sample loading control.

k ZIKV genomic RNA decay assay in Huh7 cells. The genomic RNA of a RdRp-deficient ZIKV [9] was transfected into Huh7 cells of wild type (WT) or IGF2BP1 knockout (KO), respectively. The intracellular ZIKV RNA levels were quantified relative to *GAPDH* by qPCR at different hours post transfection; the percentage relative to cells at 0 h post transfection is shown.

l In vitro translation assay of ZIKV RNA. Top, schematic for the construction of the ZIKV 5R3 reporter, where the Rluc ORF was fused with the ZIKV RNA 5'UTR and 3'UTR (5R3). R0, Rluc RNA without IGF2BP1 binding, was used as a control. Capped RNA (R0 or 5R3) combined with Firefly luciferase (Fluc) RNA (molar ratio 4:1) were incubated in Rabbit reticulocytes lysates, together with the purified IGF2BP1 protein (200 ng) or equal volume of the buffer used with IGF2BP1. The relative luciferase activities (Rluc/Fluc) were detected and calculated in the indicated conditions and normalized to R0 supplemented with the buffer used with IGF2BP1 (Buffer). ** $P < 0.01$. Two-tailed student's *t*-test.

Reference:

8. Chua R. L. et al. COVID-19 severity correlates with airway epithelium-immune cell interactions identified by single-cell analysis. *Nat. Biotechnol.* **38**, 970-979 (2020).
9. Liu Z. Y. et al. Characterization of cis-Acting RNA Elements of Zika Virus by Using a Self-Splicing Ribozyme-Dependent Infectious Clone. *J. Virol.* **91**, e00484-00417 (2017).
