# Supplementary material for: Comparison of viral RNA–host protein interactomes across pathogenic RNA viruses informs rapid antiviral drug discovery for SARS-CoV-2
Source: Cell Res. 2021 Nov 4;32(1):9–23. doi: 10.1038/s41422-021-00581-y (PMC8566969; doi:10.1038/s41422-021-00581-y)
Supplement: Supplementary file 5 — Supplementary information, Fig S5 [file 41422_2021_581_MOESM5_ESM.pdf]

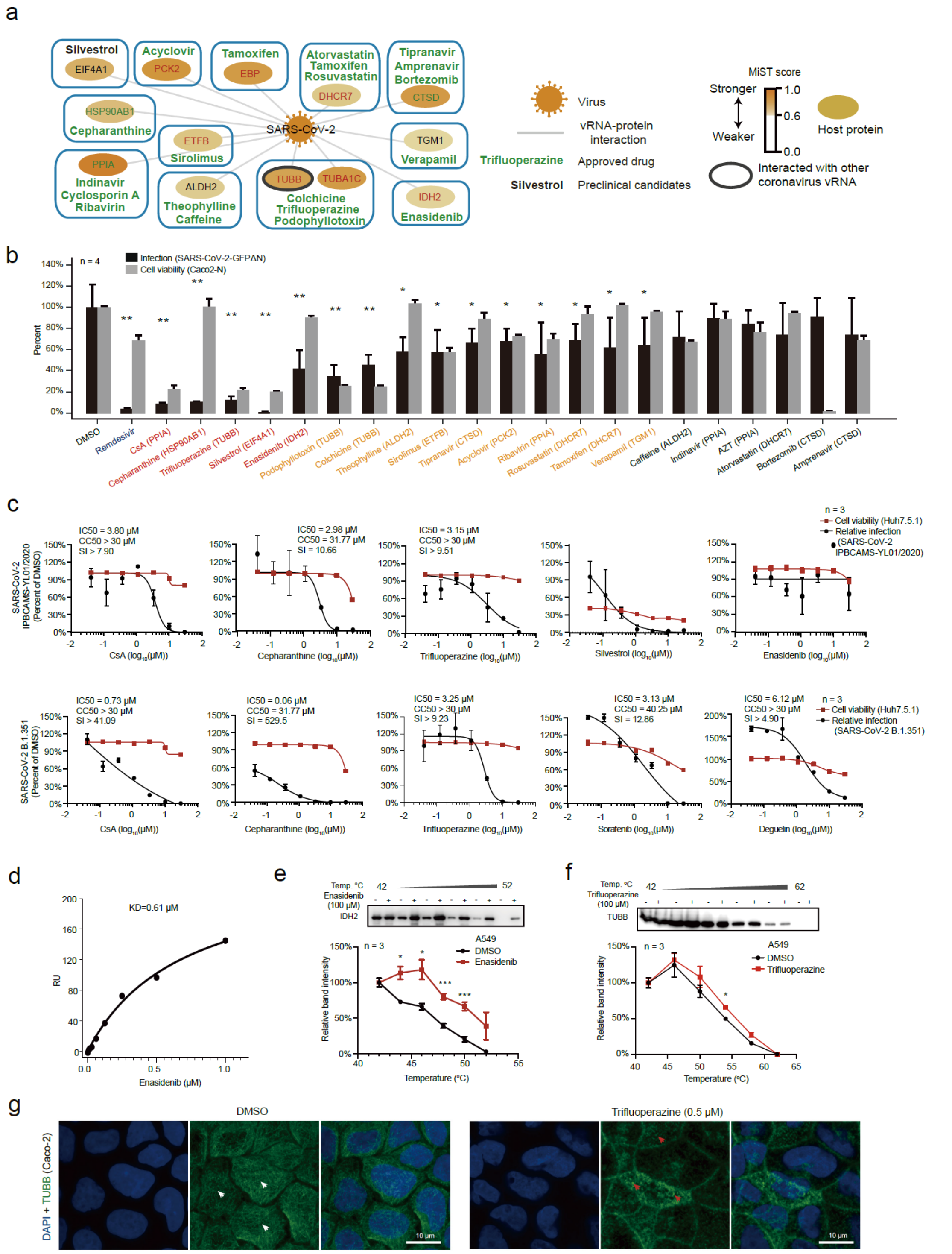

Supplementary information, Fig. S5. Testing the antiviral activities of the indicated drugs.

a FDA-approved (green) and clinical-trial (black) compounds modulating the SARS-CoV-2 vRNA interacting proteins are presented. Nodes are colored by the MiST scores

of SARS-CoV-2 vRNA interacting proteins, and protein names are colored by the up- (red) or down-regulation (green) upon SARS-CoV-2 infection [10].

b SARS-CoV-2-GFPΔN infection ratios in cells treated with the indicated drugs at a concentration of 10μM. Caco2-N cells were infected with SARS-CoV-2-GFPΔN virus (Supplementary information, Fig. S4c) for 72 h with drug treatment at day 0. Black bar, infection ratios of the SARS-CoV-2-GFPΔN. Grey bar, cell viabilities after treatment with the indicated compounds at 10μM. Data were normalized to DMSO treatment. Remdesivir was used as a positive control (2μM). Drugs selected for further validation were highlighted in red. Drugs with modest antiviral effects are highlighted in yellow. Drugs with no significant antiviral activities at a concentration of 10μM are in black. Data are means ± SD. $n = 4$ biologically independent samples. ** $P < 0.01$; * $P < 0.05$; Two-tailed student's *t*-test

c Antiviral effects of the indicated drugs against SARS-CoV-2 (IPBCAMS-YL01/2020, first row) and its VOC (B.1.351, second row) in Huh7.5.1 cells, at different drug concentrations. Red line, cell viability; Black line, infection ratio relative to the vehicle control (DMSO) group. Data are means ± SD. $n = 3$ biologically independent samples. IC50, CC50, and SI values are indicated.

d Surface Plasmon Resonance (SPR) to test the affinity of Enasidenib to IDH2. KD, association constant measured by SPR. RU, relative response unit.

e, **f** Target engagement assay of Enasidenib (**e**) and Trifluoperazine (**f**) in A549 cells. Cellular thermal shift assay (CETSA) experiment revealed Enasidenib and Trifluoperazine target IDH2 and TUBB, respectively. Top, western blot of IDH2 or TUBB protein in cells treated with Enasidenib (**e**) or Trifluoperazine (**f**), DMSO treatment was used as control. Bottom, quantification of the western blot. *** $P < 0.001$; ** $P < 0.01$; * $P < 0.05$. Two-tailed student's *t*-test.

g Immunofluorescence of TUBB containing cytoskeleton in Caco-2 cells treated with DMSO or Trifluoperazine (0.5 μM). Green, TUBB was detected using a specific antibody. Blue, nucleus stained using DAPI. Scale bar, 10 μm.

Reference:

10. Bojkova D. et al. Proteomics of SARS-CoV-2-infected host cells reveals therapy targets. *Nature* **583**, 469-472 (2020).
