# Supplementary material for: Comparison of viral RNA–host protein interactomes across pathogenic RNA viruses informs rapid antiviral drug discovery for SARS-CoV-2
Source: Cell Res. 2021 Nov 4;32(1):9–23. doi: 10.1038/s41422-021-00581-y (PMC8566969; doi:10.1038/s41422-021-00581-y)
Supplement: Supplementary file 6 — Supplementary information, Fig S6 [file 41422_2021_581_MOESM6_ESM.pdf]

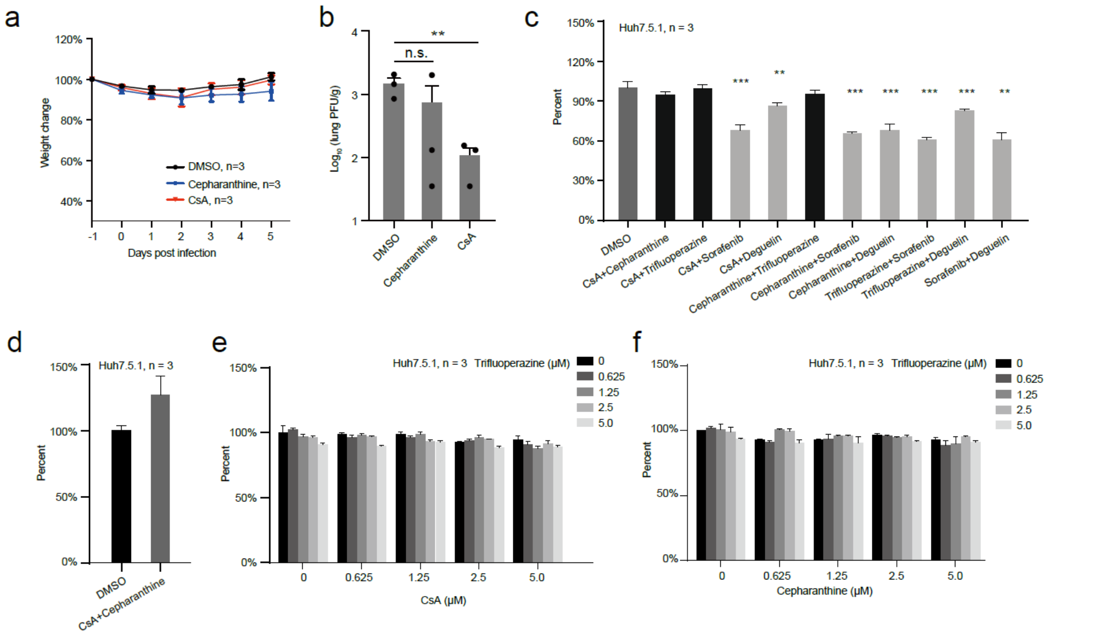

Supplementary information, Fig. S6. Evaluation of the clinical potential and pharmacological interactions of the selected drugs.

a Weight loss of hACE2 mice after infection with SARS-CoV-2. As in **Fig. 5d**, drug or vehicle was administered intranasally onto hACE2 transgenic mice. The mice were subsequently subjected to intranasal challenge with SARS-CoV-2. The body weight of vehicle- (DMSO, n = 3), Cepharanthine- (n = 3), and CsA-treated (n = 3) mice were recorded for 5 days post infection.

b Plaque assay of viral loads in mice lung tissue. Vehicle- (DMSO, n = 3), Cepharanthine- (n = 3), and CsA-treated (n = 3) mice were challenged with SARS-CoV-2. Viral loads were analyzed in the lungs of mice at 3 dpi by plaque assay. Data are means ± SEM, n = 3 independent biological samples. ** $P < 0.01$. One-tailed student's t-test.

c Cell viabilities after co-treatment with the indicated combined drugs. Huh7.5.1 cells were co-treated with concentration of 5 μM for each drug. Cell viabilities were normalized to DMSO treated cells. Black bars, cell viability > 90%. Grey bars, cell viability < 90%. Data are means ± SD. n= 3 biologically independent samples. *** $P < 0.001$; ** $P < 0.01$. Two-tailed student's t-test.

d The antiviral activity of a co-treatment comprising CsA and Cepharanthine. Huh7.5.1 cells were co-treated with CsA and Cepharanthine (5 μM each) and infected with the B.1.351 strain of SARS-CoV-2 (MOI 0.05). Cells treated with the DMSO vehicle were used as control. Viral loads in culture supernatants were quantified at 48 h post infection by qPCR (represented as percentage relative to the DMSO control treatment). Data are means ± SD. $n = 3$ biologically independent samples.

e, **f** Cell viability upon co-treatment with CsA and Trifluoperazine (**e**), or Cepharanthine and Trifluoperazine (**f**). Huh7.5.1 cells were co-treated with different concentrations of the indicated drugs. Cell viabilities were normalized to DMSO-treated cells. Data are means ± SD. $n = 3$ biologically independent samples.
